# Neuroprotective Effect of Fisetin Through Suppression of IL-1R/TLR Axis and Apoptosis in Pentylenetetrazole-Induced Kindling in Mice

**DOI:** 10.3389/fneur.2021.689069

**Published:** 2021-07-21

**Authors:** Saima Khatoon, Nidhi Bharal Agarwal, Mohammed Samim, Ozair Alam

**Affiliations:** ^1^Department of Medical Elementology and Toxicology, School of Chemical and Life Sciences, Jamia Hamdard, New Delhi, India; ^2^Centre for Translational and Clinical Research, School of Chemical and Life Sciences, Jamia Hamdard, New Delhi, India; ^3^Department of Chemistry, School of Chemical and Life Sciences, Jamia Hamdard, New Delhi, India; ^4^Department of Pharmaceutical Chemistry, School of Pharmaceutical Education and Research, Jamia Hamdard, New Delhi, India

**Keywords:** pentylenetetrazole, fisetin, interleukin, toll like receptor-4, caspase-3, epilepsy

## Abstract

Epilepsy is a complex neurological disorder, characterized by frequent electrical activity in brain regions. Inflammation and apoptosis cascade activation are serious neurological sequelae during seizures. Fisetin (3, 3′,4′,7-tetrahydroxyflavone), a flavonoid molecule, is considered for its effective anti-inflammatory and anti-apoptotic properties. This study investigated the neuroprotective effect of fisetin on experimental epilepsy. For acute studies, increasing current electroshock (ICES) and pentylenetetrazole (PTZ)-induced seizure tests were performed to evaluate the antiseizure activity of fisetin. For the chronic study, the kindling model was established by the administration of PTZ in subconvulsive dose (25 mg/kg, i.p.). Mice were treated with fisetin (5, 10, and 20 mg/kg, p.o.) to study its probable antiseizure mechanism. The kindled mice were evaluated for seizure scores. Their hippocampus and cortex were assessed for neuronal damage, inflammation, and apoptosis. Histological alterations were observed in the hippocampus of the experimental mice. Levels of high mobility group box 1 (HMGB1), Toll-like receptor-4 (TLR-4), interleukin-1 receptor 1 (IL-1R1), interleukin-1β (IL-1β), interleukin-6 (IL-6), and tumor necrosis factor-α (TNF-α) were assessed in the hippocampus and cortex by ELISA. The immunoreactivity and mRNA expressions of nuclear factor-κB (NF-κB), cyclooxygenase-2 (COX-2), cytochrome C, and caspase-3 were quantified by immunohistochemical analysis and real-time PCR. Phosphorylation ELISA was performed to evaluate AkT/mTOR (mammalian target of rapamycin) activation in the hippocampus and cortex of the kindled mice. The results showed that fisetin administration increased the seizure threshold current (STC) in the ICES test. In PTZ-induced seizures, fisetin administration increased the latency for myoclonic jerks (MJs) and generalized seizures (GSs). In the PTZ-induced kindling model, fisetin administration dose-dependently suppressed the development of kindling and the associated neuronal damage in the experimental mice. Further, fisetin administration ameliorated kindling-induced neuroinflammation as evident from decreased levels of HMGB1, TLR-4, IL-1R1, IL-1β, IL-6, and TNF-α in the hippocampus and cortex of the kindled mice. Also, the immunoreactivity and mRNA expressions of inflammatory molecules, NF-κB, and COX-2 were decreased with fisetin administration in the kindled animals. Decreased phosphorylation of the AkT/mTOR pathway was reported with fisetin administration in the hippocampus and cortex of the kindled mice. The immunoreactivity and mRNA expressions of apoptotic molecules, cytochrome C, and caspase-3 were attenuated upon fisetin administration. The findings suggest that fisetin shows a neuroprotective effect by suppressing the release of inflammatory and apoptosis molecules and attenuating histological alterations during experimental epilepsy.

## Introduction

Epilepsy is a chronic disease of the brain, conferred as one of the most prevalent neurological conditions affecting around 70 million individuals worldwide, and about 80% of them reside in developing countries (1). It is characterized by synchronized neural excitation that causes spontaneous perennial seizures (2). Currently available anti-seizure drugs (ASDs) mainly aim to modulate ion channels or neurotransmitters that eventually suppress neuronal excitability in the brain. Despite these advances, nearly 30–40% of patients with epilepsy have refractory seizures after the administration of ASDs (3) that only offer symptomatic relief and do not address the involved underlying mechanisms (4). Therefore, the development of new therapeutics that can suppress seizures by modifying the underlying pathogenesis is a major concern in drug discovery process.

Synchronous discharges during seizures followed by a period of hyperpolarization collectively lead to molecular and morphological changes within the brain (5). The hippocampus and cortex are crucial structures that have well-defined neuronal circuits involved in the genesis of the disease and are more susceptible to seizure-induced neuronal injury (6–8).

The pathophysiological mechanisms confirm that the process of epileptogenesis triggers a neuroinflammatory cascade (9). Among the proinflammatory enzymes, cyclooxygenase-2 (COX-2) is rapidly induced after inflammatory processes and plays an important role in seizure events and epileptogenesis (10). The upregulation of COX-2 following seizures activate the transcription factor nuclear factor-κB (NF-κB), which is a key regulatory kinase responsible for the release of cytokines (11, 12). The interleukin-1 receptor/Toll-like receptor (IL-1R/TLR) signaling pathway is a widely studied neuroinflammatory cascade involved in the initiation and exacerbation of seizures (13, 14). These receptors are crucial for the activation of innate immunity upon recognition of pathogen-associated molecular patterns. After a seizure insult or during frequent seizures, TLR4 gets activated by endogenous “danger signals,” usually high mobility group box 1 (HMGB1), which is released by neurons and astrocytes in the brain in response to a pathogenic injury (15). Similarly, IL-1R1 gets activated during epilepsy by interleukin-1β (IL-1β), which is released from neurons, glial cells, brain endothelium, and macrophages that have been extravasated in the brain from the blood (16, 17). Furthermore, inflammatory cytokines like interleukin-6 (IL-6) and tumor necrosis factor-α (TNF-α) are reported to be upregulated during seizures (18, 19). Additionally, the laboratory findings support that upsurge in the levels of IL-1β, IL1-Ra (interleukin1-receptor antagonist), IL-6, and TNF-α in brain tissues are associated with chronic seizures (20).

Another cell signaling cascade that is implicated in clinical and preclinical epilepsy is the hyperactivated mammalian target of the rapamycin (mTOR) pathway. It is a serine/threonine protein kinase that regulates functions such as cell survival, cellular proliferation, and inflammation (21). Studies offer a rationale that AkT/mTOR signaling pathway inhibition could be a possible target for inflammation-linked disorders (22).

Repeated seizures disrupt the mitochondrial respiratory chain and ultimately cause damage to the mitochondrial ultrastructure in the brain (23, 24). Subsequent studies have shown that prolonged seizures lead to nitrosative and oxidative stress, causing the release of cytochrome C from the mitochondria to the cytoplasm, and triggers caspase-3 activation, leading to apoptotic signaling and causing neuronal cell death in an epileptic brain (25, 26).

The last few years have seen an increase in the use of phytoconstituents in treating central nervous system (CNS) diseases due to their neuromodulatory effects. Fisetin (3,3′,4′,7-tetrahydroxyflavone) is a therapeutically active flavonoid compound that is found in fruits and vegetables, with the highest concentration found in strawberries (27). Interestingly, upon oral administration, fisetin and its active metabolite, geraldol, get rapidly circulated to the blood vessels of the brain accompanied by slower dispersion into the brain parenchyma of mice and gets localized in the nucleoli of neuronal cells (28). Several biological benefits of fisetin include anti-inflammatory, cardioprotective, hepatoprotective, anti-cancer, and anti-diabetic effects (29–33). Preclinical studies have shown that fisetin protects against traumatic brain injury (TBI), aluminum chloride-induced neurotoxicity, and methylmercury-induced neurotoxicity owing to its anti-inflammatory property (34–36). Fisetin has been reported to reduce the expression of HMGB1 and the receptor for advanced glycation end products (RAGEs) (37). Furthermore, fisetin has been reported to inhibit apoptosis and the AkT/NF-κB/mTOR pathway in laryngeal carcinoma (38). Fisetin intervention downregulates TNF-α/RIPK3 signaling to reduce hepatic inflammation in high fat diet-fed mice (39). Further, fisetin administration suppressed apoptotic proteins such as caspase-3, Bcl-2, and BAX in acute kidney injury (31). Fisetin acts through the inhibition of amyloid beta-induced neuroinflammation, neuronal loss, and synaptic distortion (40) in the hippocampus of mice.

The plethora of biological activities of fisetin suggests its probable role in targeting inflammatory and apoptotic mediators associated with the pathogenesis of seizures. On this basis, this study planned to investigate the effects of fisetin administration on acute and chronic rodent models of epilepsy. Furthermore, the effects of fisetin on inflammatory mediators, neuronal damage, and apoptotic markers involved in seizure generation and progression were assessed to understand the probable anti-seizure mechanism.

## Materials and Methods

### Animals

Male Swiss albino mice weighing from 25 to 35 g were selected for the study. They were housed in polypropylene cages in the Central Animal House facility, Jamia Hamdard (New Delhi, India) under controlled conditions (humidity: 60–70%, temperature: 25 ± 2°C, 12 h light/dark cycle). Pellet diet and water were made available to the experimental animals *ad libitum*.

### Treatment Regimen

Fisetin was procured from Cayman Chemical Co. (Ann Arbor, MI, United States). PTZ and sodium valproate (VPA) were obtained from Sigma Aldrich (St. Louis, MO, United States). Fisetin was suspended in 0.1% carboxymethylcellulose (CMC) prepared in double-distilled water. PTZ was solubilized in double-distilled water. Fisetin was administered at the doses that have been reported to inhibit the onset and progression of seizures in experimental animals in previous studies (41, 42). For acute studies, fisetin was administered in doses of 5, 10, and 20 mg/kg body weight each day by oral (p.o.) route for a week. For the chronic study, the same doses of fisetin were administered daily for 5 weeks. PTZ was administered in the dose of 25 mg/kg on alternate days by intraperitoneal (i.p.) route. The volume of 10 ml/kg was kept constant for all the administered doses.

### Acute Studies

The mice were randomly divided into groups with six mice in each group. Group I was designated as control, and groups II, III, and IV were administered fisetin in the doses of 5, 10, and 20 mg/kg/days. Group V was administered phenytoin (25 mg/kg/days) and served as the positive control group in the ICES test. Phenytoin was taken as a positive control because of its efficacy against generalized tonic–clonic seizures (43). Fisetin and phenytoin were administered for a week in the mice. On the 7th day of dosing, after 1 h of drug administration, all the experiments of acute studies were performed.

#### Increasing Current Electroshock Test

Using an electro-convulsometer, a current of 2 mA in the form of electric shock was given to each mouse using ear electrodes as a single train of pulses for 0.2 s with linearly increasing intensity of 2 mA/2 s. The seizure threshold current (STC) was recorded as the current at which tonic hind limb extension (HLE) appeared. In the absence of tonic HLE observation up to a maximum current of 30 mA, no further current was given and this cut-off value was used for further calculations (44).

#### Pentylenetetrazole-Induced Seizures

The acute study on PTZ-induced seizures was carried out according to an earlier study. Fisetin and vehicle were administered 1 h before PTZ administration. PTZ was administered in a dose of 60 mg/kg in a volume of 10 ml/kg by i.p. route to induce seizures. After PTZ injection, the mice were gently placed in Plexiglas cages. The latency to myoclonic jerks (MJ) and generalized seizures (GS) was recorded for 30-min duration (44).

### Chronic Study

The experimental mice were randomly divided into five groups consisting of eight mice in each group. Group I served as control and was administered vehicle (0.1% CMC) in addition to saline on alternate days. Group II was the vehicle + PTZ group, which received 0.1% CMC daily in addition to PTZ administered on alternate days (25 mg/kg), and served as a disease group. Groups III, IV, and V were fisetin 5 + PTZ, fisetin 10 + PTZ, and fisetin 20 + PTZ groups, which received fisetin in the doses of 5, 10, and 20 mg/kg body weight p.o. daily, respectively, with PTZ administration (25 mg/kg) on alternate days. Group VI was administered VPA in the dose of 100 mg/kg i.p. daily, and PTZ (25 mg/kg) was administered on alternate days. VPA dose was selected from the previously reported study (45). Fisetin and VPA were administered 1 h before PTZ administration. The dosing procedure was carried out for five consecutive weeks.

#### Pentylenetetrazole-Induced Kindling in Mice

After the PTZ administration, the animals were observed for 30 min to assess any seizure intensity according to the Racine score (46). The severity of seizure response was evaluated using the following score points: Stage 0: no response; Stage 1: ear and facial jerks; Stage 2: myoclonic body jerks without upright position; Stage 3: myoclonic jerks, upright position with bilateral forelimb clonus; Stage 4: tonic–clonic seizures; Stage 5: generalized tonic–clonic seizures, loss of postural control. The mice were considered fully kindled when they showed a seizure score of 4 on three consecutive PTZ injections. The cumulative seizure score of each group was calculated on the 7th, 14th, 21st, 28th, and 35th day.

#### Brain Processing and Tissue Preparation

For Nissl staining and immunohistochemical analysis, the mice were anesthetized and perfused with 0.9% saline followed by 4% paraformaldehyde. Following perfusion, the brains were isolated and immersed with 4% paraformaldehyde at 4°C for 4 h, embedded in paraffin wax followed by cutting of coronal sections (4 μm) and mounted on polylysine-treated slides. For biochemical assays, the mice were anesthetized, and the brain tissues (the hippocampus and the cortex) were removed and immediately stored at −20°C. For RNA extraction, the hippocampi and cortices were removed from mice brain in dry ice and stored at −80°C until further processing.

#### Nissl Staining

Paraffin sections were de-waxed and rehydrated through a series of washes: xylol (two times for 10 min), 100% ethanol (two times for 5 min), 95% ethanol (one time for 5 min), 90% ethanol (one time for 5 min), 80% ethanol (one time for 3 min), 70% ethanol (one time for 3 min), and lastly in distilled water (5 min). Sections were stained in 0.1% cresyl violet solution (dissolved in 0.01% glacial acetic acid) at 37°C for 10 min, rinsed rapidly in distilled water, and differentiated in 95% ethyl alcohol (30 s). The neuronal damage was analyzed using a microscope (EVOS FL Cell Imaging System, Thermo Fisher Scientific, Waltham, MA, United States).

#### Immunohistochemistry

Immunoreactivity of NF-κB, COX-2, cytochrome C, and caspase-3 was carried out according to the standard protocol by Ali et al. (7). Briefly, the tissue sections obtained were rehydrated in xylene followed by graded ethanol solutions. Then, the sections were incubated for 10 min with 3% H_2_O_2_ to eliminate endogenous peroxidase activity and washed with tris-buffered saline (TBS) three times (2 min each). To diminish non-specific binding of antibodies during the staining; the slides were blocked using 5% bovine serum albumin (BSA) in TBS for a duration of 2 h. The sections were then incubated with one of the following polyclonal rabbit antibodies: NF-κB, COX-2, caspase-3, and cytochrome C (Elabscience, catalog number: E-AB-32232, E-AB-17010, E-AB-13815, and E-AB-36241, respectively) at a concentration of 1 μg/ml containing 5% BSA in TBS and incubated at 4°C overnight. After washing the slides with TBS (three times, 2 min each), the sections were incubated with goat anti-rabbit secondary antibody (Dako, Copenhagen, Denmark). Then, the sections were washed with TBS and incubated for 5–10 min in a solution of 0.02% diaminobenzidine (Dako, Copenhagen, Denmark). Counter-staining was performed with hematoxylin (47). The pyramidal cells of the hippocampal CA1 region were selected and analyzed for optical density (OD). The images were acquired using a light microscope (EVOS FL Cell Imaging System, Thermo Fisher Scientific, Waltham, MA, United States) and processed using ImageJ 1.47, NIH, USA software.

#### Real-Time PCR

Total RNA was extracted from the hippocampus and cortex tissues using Trizol reagent (Invitrogen, Waltham, MA, United States). RNA yield and purity were assessed by nanodrop (Thermo Fisher Scientific, Waltham, MA, United States). RNA from each sample was reverse transcribed to cDNA using commercially available Verso cDNA Synthesis Kit (Thermo Fisher Scientific, Waltham, MA, United States) as per the instructions of the manufacturer. Real-time PCR conditions used are as followed: 95°C (5 min; initial denaturing), 40 cycles of 94°C (15 s; denaturing), 63°C (1 min; annealing) and 72°C (30 s; extension). Multi-well plate-96 was run on Roche Light Cycler 480 using a 5-μM forward primer, 0.5-μM reverse primer, 1.5 μl of cDNA, 5 μl of SyBr Green Fast, a total of 10 μl reaction using nuclease-free water. The fold change expression for a particular gene was calculated using a comparative 2^−ΔΔCt^ method (48). As a reference standard, mouse GAPDH was used. The mean values of each group were calculated, and the change in the number of folds in comparison with control was noted. The primer sequences were taken from the published reports (49, 50) and are as follows: NF-κB (forward: 5′-ACGACATTGAGGTTCGGTTC-3′, reverse: 5′-ATCTTGTGATAGGGCGGTGT-3′); COX-2 (forward: 5′- GCAGATGACTGCCCAACTC-3′, reverse: 5′-GGAGGCTAAGTGGAGCTGAC-3′); cytochrome C (forward: 5′-AGGCTGCTGGATTCTCTTACAC-3′, reverse: 5′-CAGGGATGTACTTTTTGGGATT-3′); caspase-3 (forward: 5′-GGAGCAGTTTTGTGTGTGTGAT-3′, reverse: 5′-GAAGAGTTTCGGCTTTCCAGT-3′); and GAPDH (forward: 5′-ATCCTGTAGGCCAGGTGATG-3′, reverse: 5′-TATGCCCGAGGACAATAAGG-3′).

#### Enzyme-Linked Immunosorbent Assay (ELISA)

Commercially available ELISA kits from Bioassay Technology Laboratory (Birmingham, United Kingdom) were used to quantitatively assess the levels of HMGB1, TLR-4, IL-1β, IL-1R1, IL-6, and TNF-α (catalog numbers: E0523Mo, E1663Mo, E0192Mo, E2665Mo, E0049Mo, and E0117Mo, respectively) in the hippocampus and cortex tissues of the experimental animals. Phosphorylation ELISA kits from RayBiotech (Peachtree Corners, GA, United States) were used to estimate the levels of total and phosphorylated forms of AkT and mTOR (catalog numbers: PEL-AKT-S473-T and PEL-mTOR-S2448-T, respectively).

### Statistical Analysis

The experimental results are expressed as mean ± SEM. The parameters were analyzed by one-way ANOVA followed by Tukey's multiple comparison test except for the ICES test and PTZ-induced seizures that were analyzed by Dunnett's multiple comparison test using the GraphPad Prism 6 software. *p* < 0.05 was considered to be statistically significant.

## Results

### Effect of Fisetin Administration on ICES Test

Group II (fisetin 5 mg/kg, p.o.) was unable to show any significant increase in STC in the experimental mice when compared with control (group I). Groups III and IV, which were administered fisetin in doses 10 and 20 mg/kg/days, increased STC significantly (*p* < 0.01 and *p* < 0.001, respectively) in the mice as compared with the control group. Moreover, the STC recorded in group V, which was administered phenytoin 25 mg/kg/days (positive control group), was found to be significantly increased (*p* < 0.001) when compared with the control group and was non-significant when compared with groups III and IV. This represents the protection of fisetin in doses 10 and 20 mg/kg against HLE, which are comparable with phenytoin (Table 1).

**Table 1:** Effect of fisetin administration on seizure threshold current on ICES test in experimental mice.

**Group**	**Treatment**	**Dose (mg/kg)**	**Seizure threshold current (STC) mA**
I	Control	10 ml	16.33 ± 1.20
II	Fisetin (5 mg/kg)	5	17.00 ± 0.86
III	Fisetin (10 mg/kg)	10	23.67 ± 0.95^a^
IV	Fisetin (20 mg/kg)	20	24.00 ± 1.15^b^
V	Phenytoin	25	24.33 ± 1.58^c^

*Fisetin pre-treatment significantly increased the STC in a dose-dependent manner when compared with control. Data were analyzed by one-way ANOVA followed by Dunnett's multiple comparison test (n = 6)*.

a *P < 0.01 for control vs. fisetin 10 mg/kg*;

b *P < 0.001 for control vs. fisetin 20 mg/kg*;

c *P < 0.001 for control vs. phenytoin*.

### Effect of Fisetin Administration on PTZ-Induced Seizures

In PTZ-induced seizures, fisetin 5, 10, and 20 mg/kg was administered for seven consecutive days followed by PTZ (60 mg/kg) administration on the seventh day. Group II, which was administered fisetin 5 mg/kg, failed to show any significant increase in latency to MJ and GS as compared with group I (control). Groups III and IV mice were administered fisetin (10 and 20 mg/kg/days) and showed a significant increase (*p* < 0.001) in the latency to -MJ and -GS when compared with group I. Since PTZ-induced seizures represent absence seizures (51), the findings show that fisetin has dose-dependent efficacy against absence seizures in the experimental mice (Table 2).

**Table 2:** Effect of fisetin administration on PTZ-induced seizures in experimental mice.

**Group**	**Treatment**	**Dose (mg/kg)**	**Latency to myoclonic jerks (sec)**	**Latency to generalized seizures (sec)**
I	Control	10 ml	69.83 ± 1.72	101.33 ± 3.53
II	Fisetin (5 mg/kg)	5	93.49 ± 2.19	156.17 ± 3.57
III	Fisetin (10 mg/kg)	10	748.50 ± 14.78^a^	1093.70 ± 14.57^a^
IV	Fisetin (20 mg/kg)	20	762.30 ± 14.51^b^	1063.20 ± 27.99^b^

*Fisetin administration significantly increased the latency for myoclonic jerks and generalized seizures in a dose-dependent manner. Data were analyzed by one-way ANOVA followed by Dunnett's multiple comparison test (n = 6)*.

a *P < 0.001 for control vs. fisetin 10 mg/kg and*

b *P < 0.001 for control vs. fisetin 20 mg/kg*.

### Effect of Fisetin Administration on PTZ-Induced Kindling

**Figure 1:**
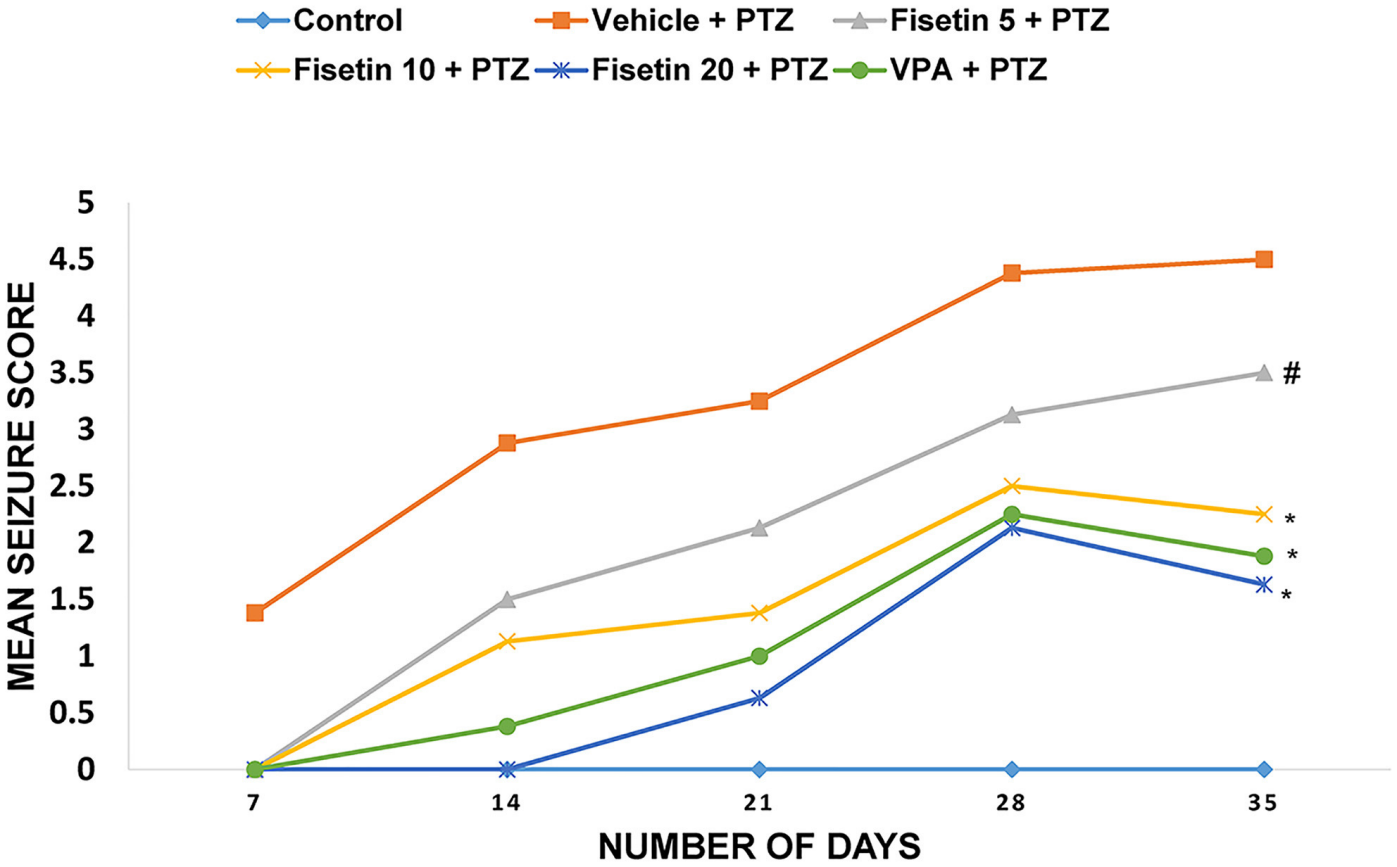
Effect of fisetin administration on mean seizure scores in kindled mice on 7th, 14th, 21st, 28th, and 35th day of dosing. Fisetin administration significantly decreased the mean seizure score and increased the latency for seizure generation. Fisetin administration in doses (10 and 20 mg/kg, p.o.) inhibited the development of fully kindled seizures. Fisetin administration prevented mortality during the kindling procedure. Data were analyzed by one-way ANOVA followed by Tukey's multiple comparison test (*n* = 8) and expressed as mean ± SEM. ^#^*p* < 0.05 as compared with vehicle + PTZ group and ^*^p < 0.001 as compared with vehicle + PTZ group.

The anticonvulsant effect of fisetin was explored in PTZ-induced kindling, which is a chronic model of generalized seizures (Figure 1, Supplementary Table 1). On the 7th, 14th, 21st, 28th, and 35th days, mean seizure scores of each group were calculated. Vehicle + PTZ group achieved stage 4 seizure by day 21 showing generalized tonic–clonic seizures. The mean seizure score was 4.5 ± 0.19 in the vehicle + PTZ group after 5 weeks. In groups fisetin 5 + PTZ, fisetin 10 + PTZ, and fisetin 20 + PTZ, the mean seizure scores were reduced to 3.5 ± 0.42, 2.25 ± 0.25, and 1.63 ± 0.18, respectively, on day 35. Further, in VPA + PTZ group, the mean seizure score was reduced to 1.88 ± 0.13 on day 35. Treatment with all three doses of fisetin increased the number of days required for stimulation for various stages of seizures. Also, all experimental mice in the vehicle + PTZ group were fully kindled. In the fisetin 5 + PTZ group, 40% of mice were fully kindled. However, in fisetin 10 + PTZ, fisetin 20 + PTZ, and VPA + PTZ groups, there were no appearance of stage 4 and 5 seizures (no full kindling obtained). In addition to this, mortality was reported in vehicle + PTZ and fisetin 5 + PTZ treated groups (two and one mice, respectively), whereas fisetin 10 + PTZ, fisetin 20 + PTZ, and VPA + PTZ groups showed no mortality in experimental animals.

### Effect of Fisetin Administration on Histopathological Changes in Kindled Mice

**Figure 2:**
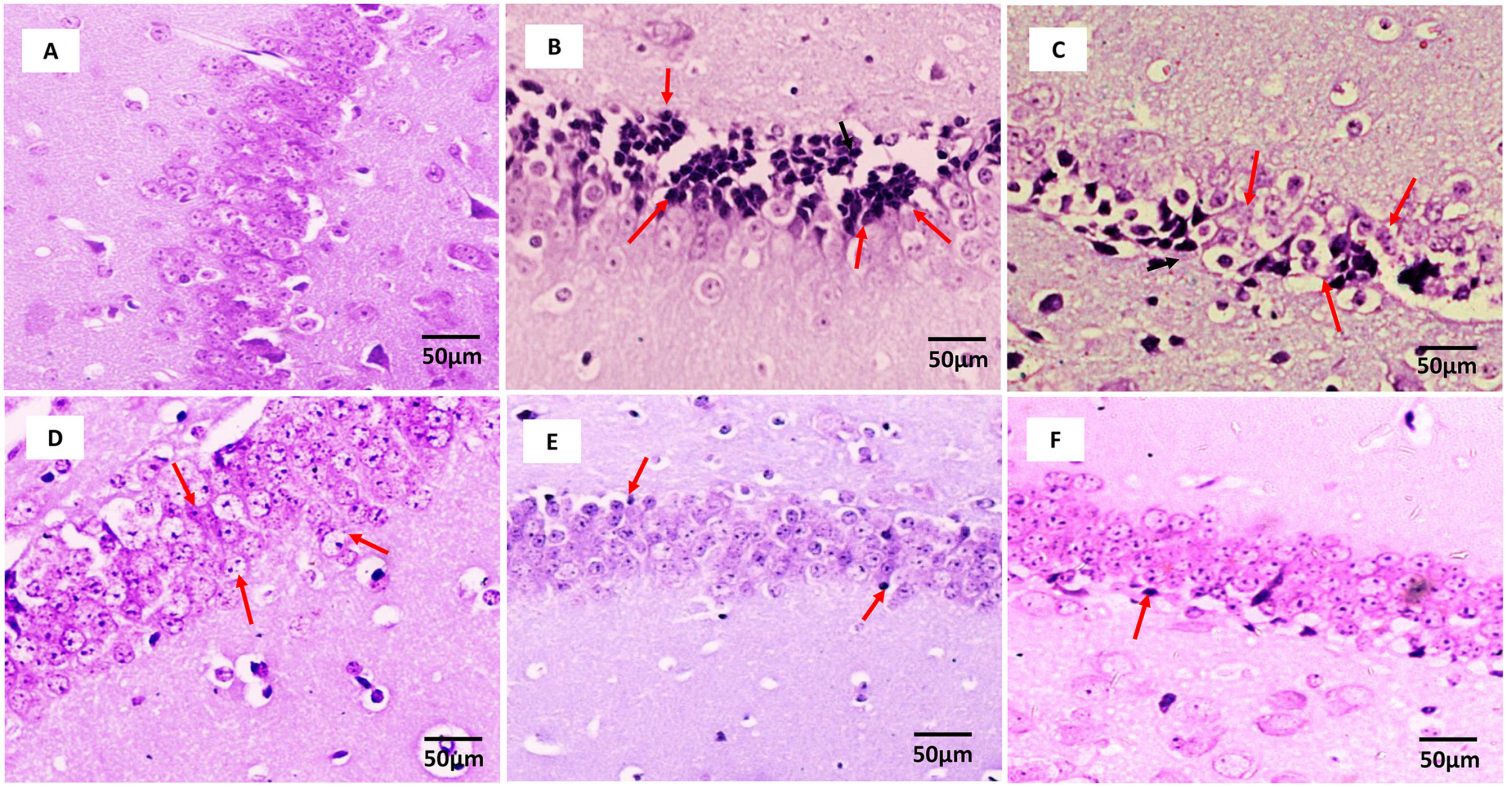
Photomicrographs showing hippocampus of mice brain after treatment with vehicle, vehicle + PTZ, fisetin 5 + PTZ, fisetin 10 + PTZ, fisetin 20 + PTZ, and VPA + PTZ. Nissl staining was performed to illustrate structural changes and neuronal damage in the CA1 region of the hippocampus (40X). **(A)** In the control group, neurons are properly arranged and display proper edges with clearly visible cytoplasm. **(B)** Vehicle + PTZ group shows highly deranged hippocampal neurons which are loose with deeply stained cytoplasm. **(C)** Fisetin 5 + PTZ group mice brain showing distorted pyramidal cell layer and neurons with dark-colored cytoplasm as shown by arrows. **(D)** Fisetin 10 + PTZ group shows almost normal structural morphology similar to the control group as shown by arrows. **(E)** Fisetin 20 + PTZ group showed healthy cellular morphology of neuronal cells similar to the control group. **(F)** VPA + PTZ group showing normal histology of neuronal cells similar to groups **(D,E)**.

Figure 2 (Supplementary Figure 1) shows the microscopic assessment of the hippocampus sections taken from the experimental groups. The low seizure threshold and the generation of seizures in the hippocampus make it the most vulnerable region during epileptogenesis. Morphological features of neurons in the hippocampus region of the control group and fisetin treated groups include a highly dense pyramidal layer with intact and clear structures. On the contrary, neuronal damage was observed in the vehicle + PTZ group. The damage was characterized by disintegrated neurons in the CA1 region marked with pyknotic and atrophic cytoplasmic vacuolation, chromatin clumping, and uneven shape. In contrast, fisetin administration in two doses, 10 and 20 mg/kg, p.o. preserved the morphology of neurons in the hippocampus. Also, VPA administration showed intact hippocampal morphology and cell number. The results indicate that fisetin could dose-dependently suppress the neuronal damage in mice, which is a hallmark associated with seizures. The histopathological alterations are summarized in Table 3.

**Table 3:** Effect of fisetin administration on histopathology of hippocampal CA1 region of experimental mice.

**Pathological alterations**	**Group**					
	**Control**	**Vehicle + PTZ**	**Fisetin 5 + PTZ**	**Fisetin 10 + PTZ**	**Fisetin 20 + PTZ**	**VPA + PTZ**
Degeneration of neurons	–	++	++	+	–	–
Vacuolation	–	++	+	–	–	–
Loss in cell numbers	–	++	+	–	–	–

*Scoring criteria: –no noticeable alterations, + mild alterations, ++ severe alterations*.

### Effect of Fisetin Administration on HMGB1, TLR-4, IL-1β, IL-1R1, IL-6, and TNF-α Levels in the Hippocampus and Cortex of Experimental Mice

**Figure 3:**
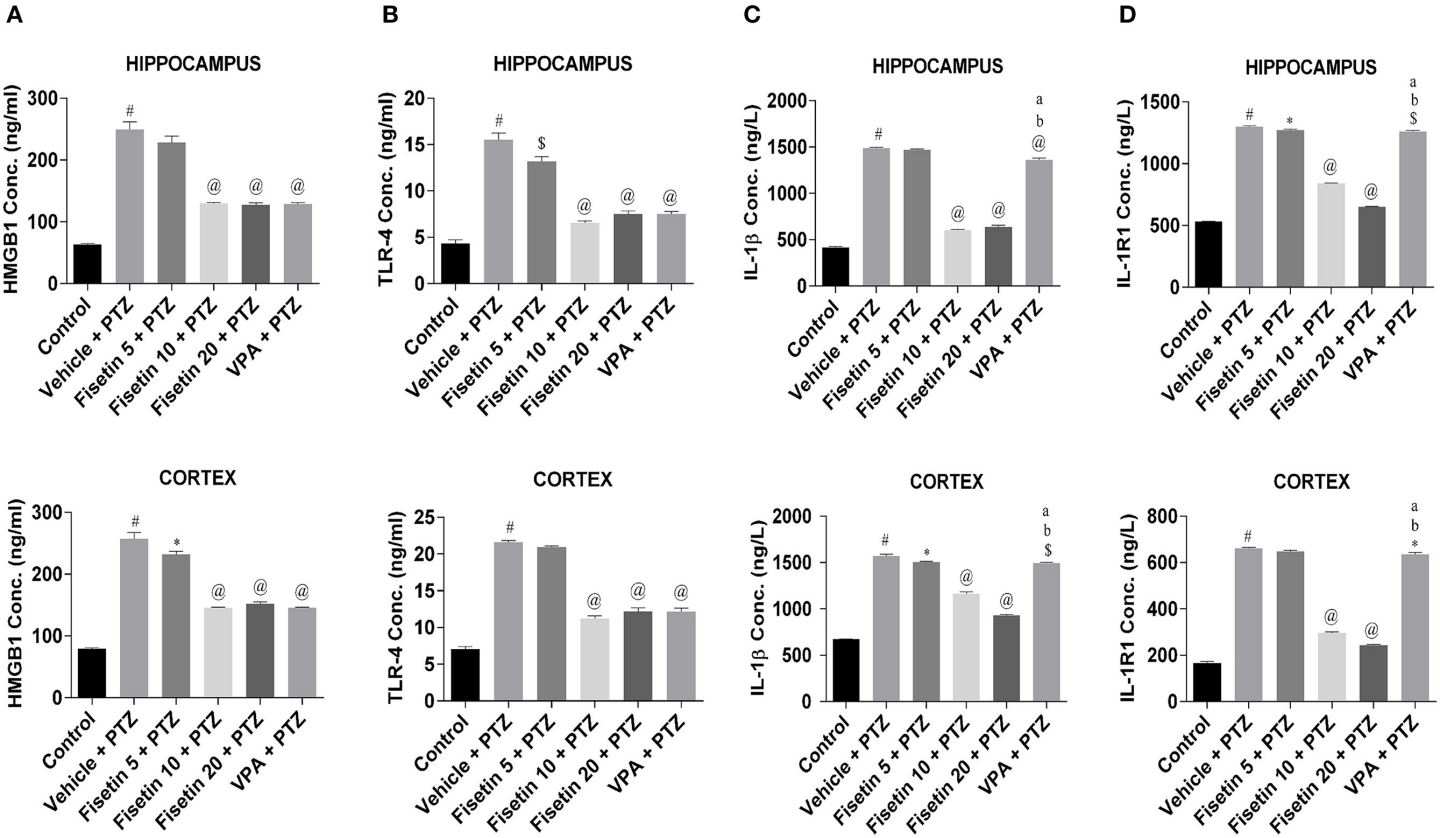
Effect of Fisetin Administration on HMGB1, TLR-4, IL-1b, and IL-1R1 Levels in the Hippocampus and Cortex of Experimental Mice. The concentrations were measured in the hippocampus and cortex of experimental animals using ELISA kits. Fisetin administration significantly lowered the levels of cytokines in a dose-dependent manner in the hippocampus and cortex tissues. Data were analyzed by one-way ANOVA followed by Tukey's multiple comparison test (*n* = 6) and expressed as mean ± SEM. ^#^*p* < 0.001 as compared with control; *p < 0.05 as compared with vehicle + PTZ group; ^$^*p* < 0.01 as compared with vehicle + PTZ group; ^@^*p* < 0.001 as compared with vehicle + PTZ group; ^a^*p* < 0.001 as compared with fisetin 10 + PTZ group, and ^b^*p* < 0.001 as compared to fisetin 20 + PTZ group.

**Figure 4:**
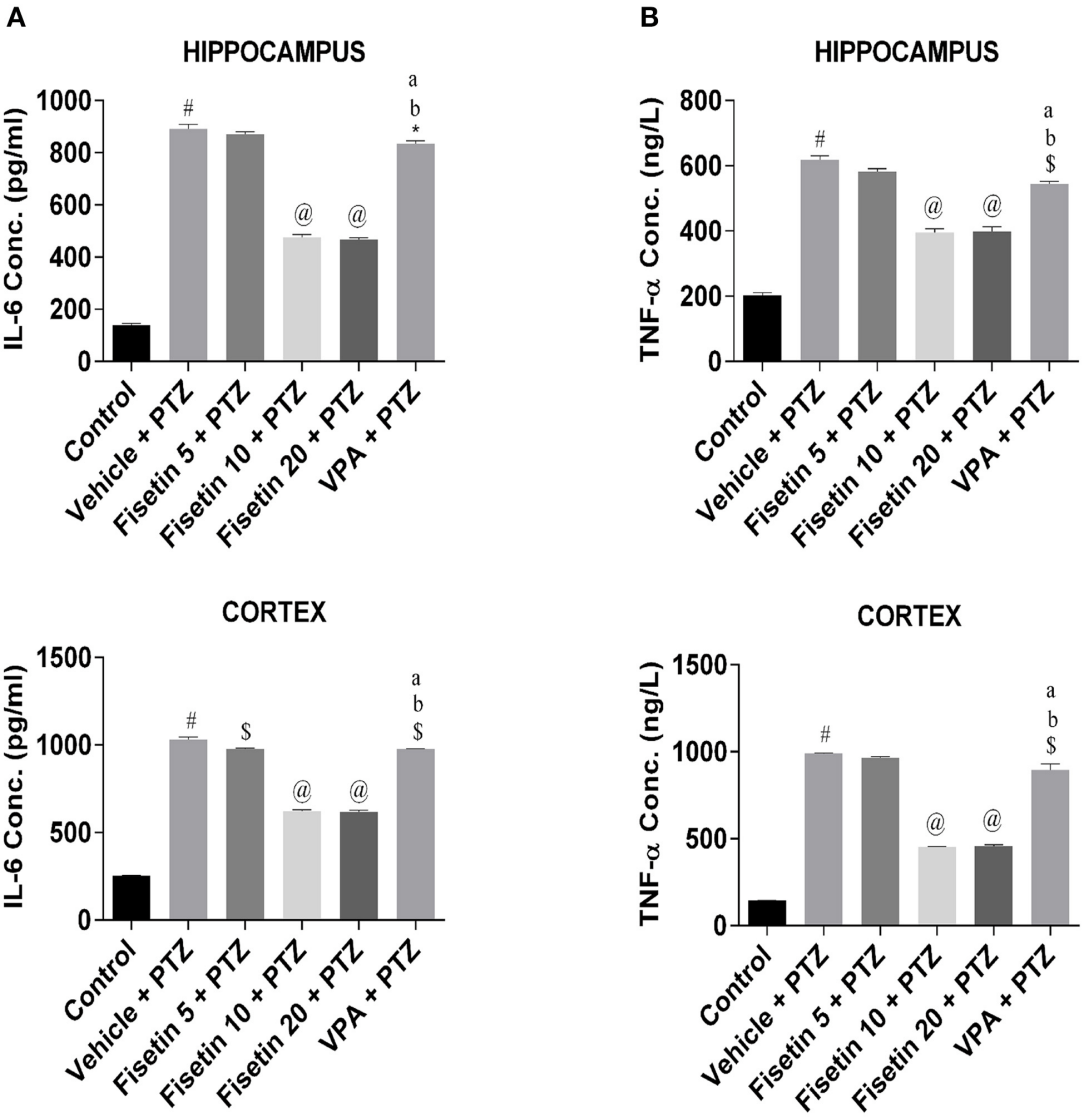
Effect of Fisetin Administration on IL-6 and TNF-a Levels in the Hippocampus and Cortex of Experimental Mice. **(A)** IL-6 and **(B)** TNF-α in the hippocampus and cortex of kindled mice. The concentrations were measured in hippocampus and cortex of experimental animals using ELISA kits. Fisetin administration significantly lowered the levels of cytokines in a dose-dependent manner in the hippocampus and cortex tissues. Data were analyzed by one-way ANOVA followed by Tukey's multiple comparison test (*n* = 6) and expressed as mean ± SEM. ^#^*p* < 0.001 as compared with control; *p < 0.05 as compared with vehicle + PTZ group; ^$^*p* < 0.01 as compared with vehicle + PTZ group; ^@^*p* < 0.001 as compared with vehicle + PTZ group; ^a^*p* < 0.001 as compared with fisetin 10 + PTZ group and ^b^*p* < 0.001 as compared with fisetin 20 + PTZ group.

Levels of inflammatory cytokines in the hippocampus and cortex of the experimental animals were estimated using ELISA kits (Figures 3, 4; Supplementary Tables 2–7). The concentration of HMGB1, TLR-4, IL-1β, IL-1R1, IL-6, and TNF-α in the hippocampus and cortex of the vehicle + PTZ group was significantly elevated (*p* < 0.001) as compared with the control group. The fisetin 5 mg/kg, p.o. + PTZ treated group was unable to show any significant decrease in the level of HMGB1 in the hippocampus but was significantly lowered in the cortex (*p* < 0.05) when compared with the vehicle + PTZ group. TLR-4 concentration was decreased in the hippocampus (*p* < 0.01) of the fisetin 5mg/kg, p.o. + PTZ group. Both HMGB1 and TLR-4 levels were significantly decreased in hippocampi and cortex tissues of the fisetin 10 mg/kg, p.o. + PTZ, fisetin 20 mg/kg, p.o. + PTZ, and VPA + PTZ groups (*p* < 0.001) when compared with the vehicle + PTZ group. Further, the fisetin 5 + PTZ group showed significantly lower (*p* < 0.05) levels of IL-1β in the cortex in comparison with the vehicle + PTZ group. In the fisetin 10 mg/kg, p.o. + PTZ and fisetin 20 mg/kg, p.o. + PTZ groups, the IL-1β level was significantly reduced (*p* < 0.001) when compared with the vehicle + PTZ group. IL-1β was significantly decreased in the hippocampus and cortex of the VPA + PTZ group (*p* < 0.001 and *p* < 0.01, respectively) as compared with the vehicle + PTZ group. However, the IL-1β concentration was found to be significantly increased in the hippocampus and cortex of the VPA + PTZ group (*p* < 0.001) when compared with the fisetin 10 mg/kg, p.o. + PTZ and fisetin 20 mg/kg, p.o. + PTZ groups. The levels of IL-1R1 were significantly decreased in the hippocampus and cortex of fisetin 10 mg/kg, p.o.+ PTZ and fisetin 20 mg/kg, p.o. + PTZ groups as compared to vehicle + PTZ group (*p* < 0.001). Also, IL-1R1 concentration was found to be significantly reduced in the hippocampus and cortex of the VPA + PTZ group (*p* < 0.01 and *p* < 0.05, respectively) as compared with the vehicle + PTZ group. However, the concentration of IL-1R1 was significantly higher in the VPA + PTZ group when compared with the fisetin 10 mg/kg, p.o. + PTZ and fisetin 20 mg/kg, p.o. + PTZ groups (*p* < 0.01). IL-6 concentration was significantly lower in the cortex of the fisetin 5 mg/kg, p.o. + PTZ group (*p* < 0.01) when compared with the PTZ group. Further, the concentrations of IL-6 and TNF-α were significantly lower in the hippocampus and cortex of the fisetin 10 mg/kg, p.o. + PTZ and fisetin 20 mg/kg, p.o. + PTZ groups when compared with the vehicle + PTZ group (*p* < 0.001). However, in the VPA + PTZ group, the levels of IL-6 and TNF-α were found to be significantly elevated in the hippocampus and cortex when compared with the fisetin 10 mg/kg, p.o. + PTZ and fisetin 20 mg/kg, p.o. + PTZ groups (*p* < 0.001).

### Effect of Fisetin Administration on NF-κB and COX-2 in the Hippocampus and Cortex of Experimental Mice

**Figure 5:**
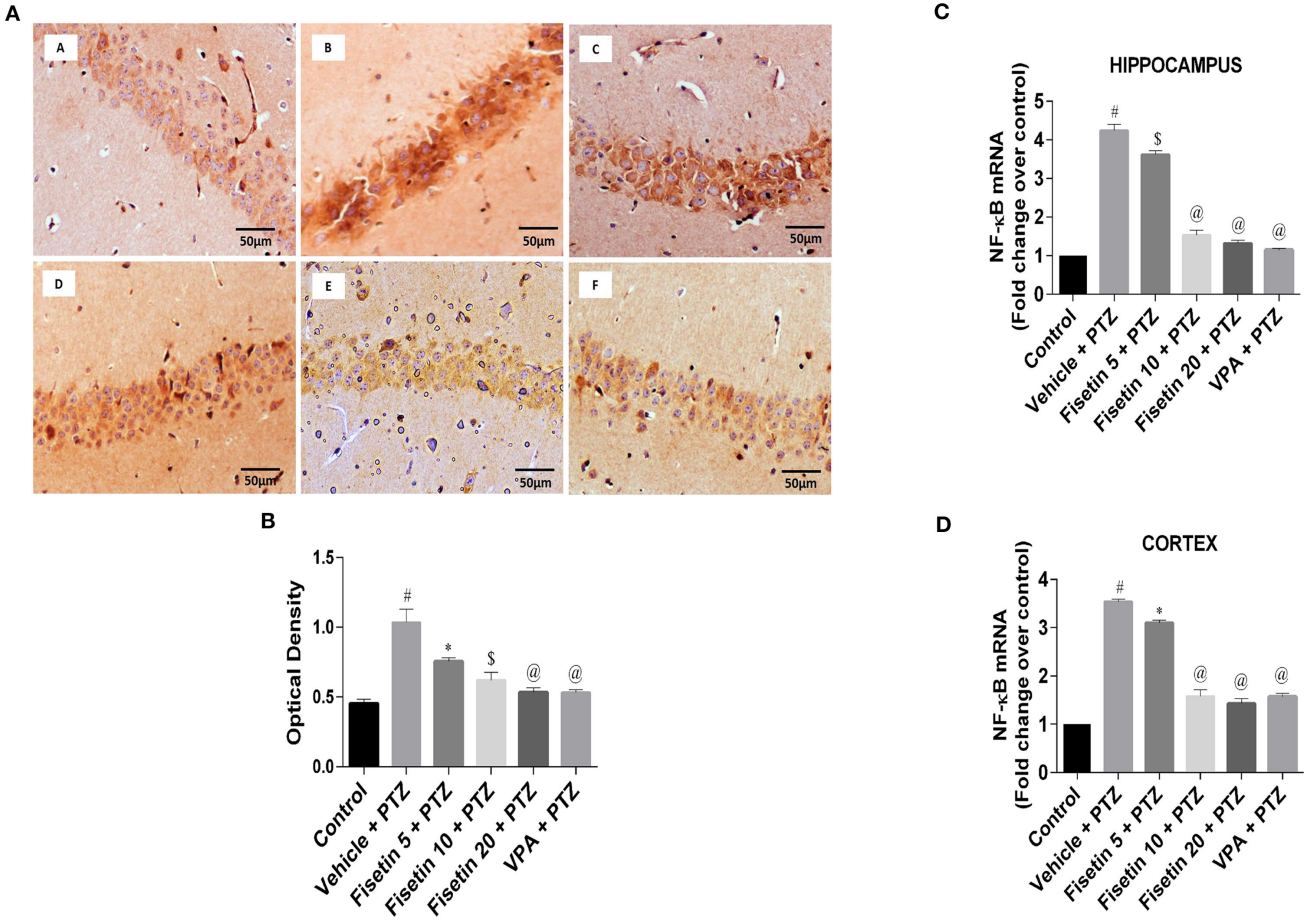
Effect of Fisetin Administration on NF-κB in the Hippocampus and Cortex of Experimental Mice. **(A)** Photomicrographs of the hippocampus in different experimental groups are shown (40X). (A) Control, (B) vehicle + PTZ, (C) fisetin 5 + PTZ, (D) fisetin 10 + PTZ, (E) fisetin 20 + PTZ, and (F) VPA + PTZ. Fisetin treatment resulted in reduced immunoreactivity of NF-κB protein in hippocampal neurons compared with the vehicle + PTZ group. **(B)** Quantitative estimation of NF-κB immunostaining (*n* = 3). **(C)** mRNA expression of NF-κB in the hippocampus (*n* = 5). **(D)** mRNA expression of NF-κB in the cortex (*n* = 5). Data were analyzed by one-way ANOVA followed by Tukey's multiple comparison test and expressed as mean ± SEM. ^#^*p* < 0.001 as compared with control group; *p < 0.05 as compared with vehicle + PTZ group; ^$^*p* < 0.01 as compared to vehicle + PTZ group, and ^@^*p* < 0.001 as compared with vehicle + PTZ group.

Figures 5A,B (Supplementary Figure 2; Supplementary Table 8) show the immunostaining of NF-κB in the different experimental groups. The vehicle + PTZ group showed significantly elevated immunoreactivity relative to control (*p* < 0.001). Fisetin 5 mg/kg, p.o. + PTZ showed significantly lowered expression of NF-κB (*p* < 0.05) when compared with the optical density of the control group. Further, in the fisetin 10 mg/kg, p.o. + PTZ, fisetin 20 mg/kg, p.o. + PTZ, and VPA + PTZ groups, the immunoreactivity was lower when compared with the vehicle + PTZ group in a significant manner (*p* < 0.01, *p* < 0.001, and *p* < 0.001, respectively). Figures 5C,D (Supplementary Table 8) show that mRNA expressions of NF-κB in the hippocampus and cortex of the vehicle + PTZ group were significantly increased (*p* < 0.001) as compared with the control group. However, the administration of fisetin 5 mg/kg, p.o. significantly attenuated the increase in NF-κB in the hippocampus and cortex of the kindled mice (*p* < 0.01 and *p* < 0.05, respectively) as compared with the vehicle + PTZ group. Fisetin (10 and 20 mg/kg, p.o.) and VPA administration in the kindled mice resulted in a significant decrease (*p* < 0.001) in mRNA expression levels of NF-κB as compared with the vehicle + PTZ group in both hippocampus and cortex tissues of the experimental mice.

**Figure 6:**
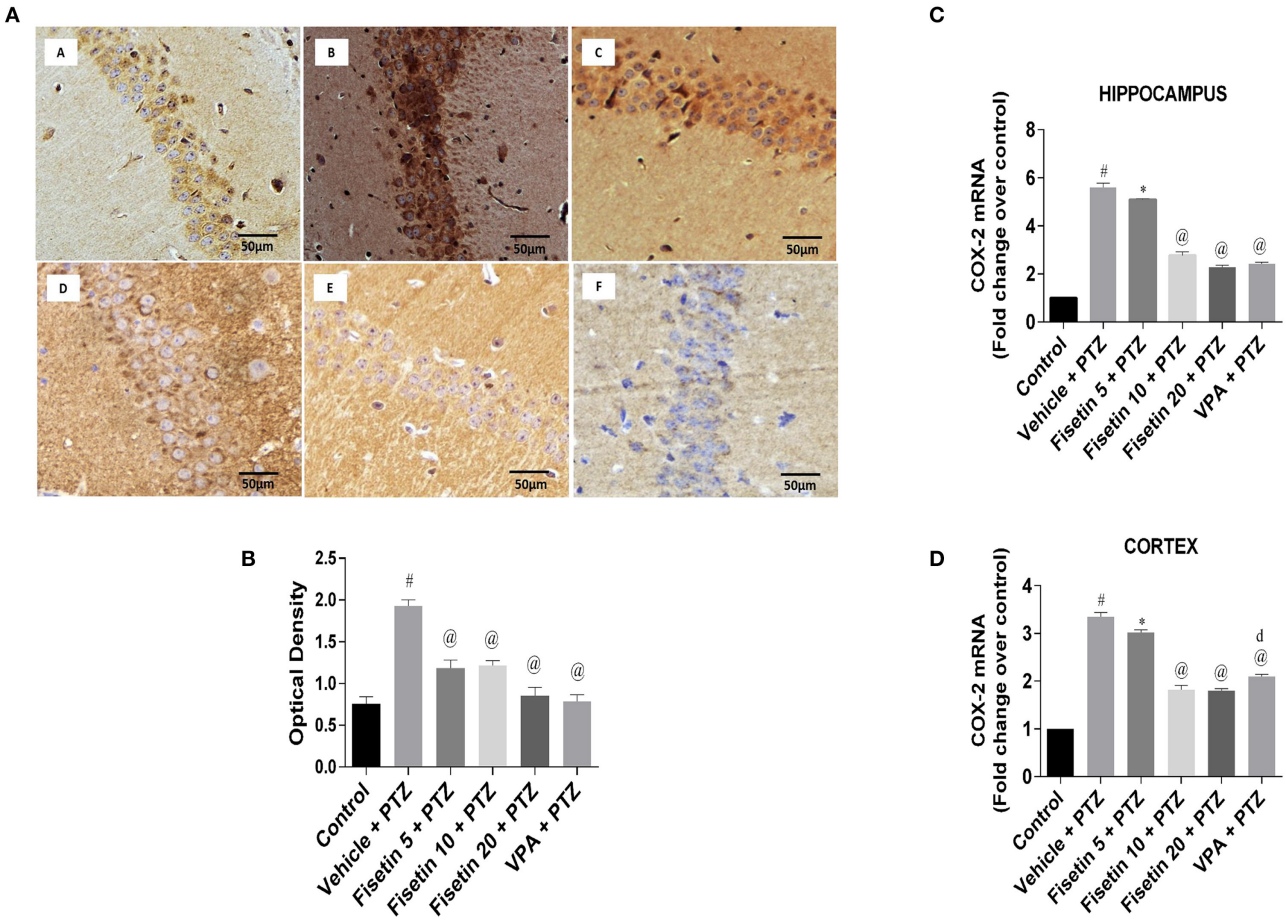
Effect of Fisetin Administration on COX-2 in the Hippocampus and Cortex of Experimental Mice. **(A)** Photomicrographs of the hippocampus in different groups are shown (40X). (A) Control, (B) vehicle + PTZ, (C) fisetin 5 + PTZ, (D) fisetin 10 + PTZ, (E) fisetin 20 + PTZ, and (F) VPA + PTZ. Fisetin administration resulted in reduced immunoreactivity of COX-2 protein in hippocampal neurons as compared with vehicle + PTZ group. **(B)** Quantitative estimation of COX-2 immunostaining (*n* = 3). **(C)** mRNA expression of COX-2 in the hippocampus (*n* = 5). **(D)** mRNA expression of COX-2 in the cortex (*n* = 5). Data were analyzed by one-way ANOVA followed by Tukey's multiple comparison test and expressed as mean ± SEM. ^#^*p* < 0.001 as compared with control group; ^*^p < 0.05 as compared to vehicle + PTZ group; ^@^*p* < 0.001 as compared with vehicle + PTZ group, and ^d^*p* < 0.05 as compared with fisetin 20 + PTZ group.

Figures 6A,B (Supplementary Figure 3; Supplementary Table 9) show the immunostaining of COX-2 in the different experimental groups. The vehicle + PTZ group showed significantly increased immunoreactivity specified by intense brown staining relative to the control mice (*p* < 0.001). The fisetin 5 mg/kg, p.o. + PTZ, fisetin 10 mg/kg, p.o. + PTZ, fisetin 20 mg/kg, p.o. + PTZ and VPA + PTZ groups showed decreased immunoreactivity (*p* < 0.001) as compared with the vehicle + PTZ group. The mRNA expressions of COX-2 in the hippocampus and cortex are depicted in Figures 6C,D (Supplementary Table 9). In the vehicle + PTZ group, the fold change expression of COX-2 was significantly increased in both hippocampus and cortex in a significant manner (*p* < 0.001) when compared with the control group. COX-2 mRNA expression was significantly reduced in the hippocampus and cortex of the fisetin 5 mg/kg, p.o. + PTZ group (*p* < 0.05) as compared with the vehicle + PTZ group. Furthermore, the fisetin 10 mg/kg, p.o. + PTZ, fisetin 20 mg/kg, p.o. + PTZ, and VPA + PTZ groups showed lowered mRNA expressions of COX-2 in the hippocampus and cortex tissues (*p* < 0.001) as compared with the vehicle + PTZ group.

### Effects of Fisetin Administration on AkT/mTOR Cascade in the Hippocampus and Cortex of Experimental Mice

**Figure 7:**
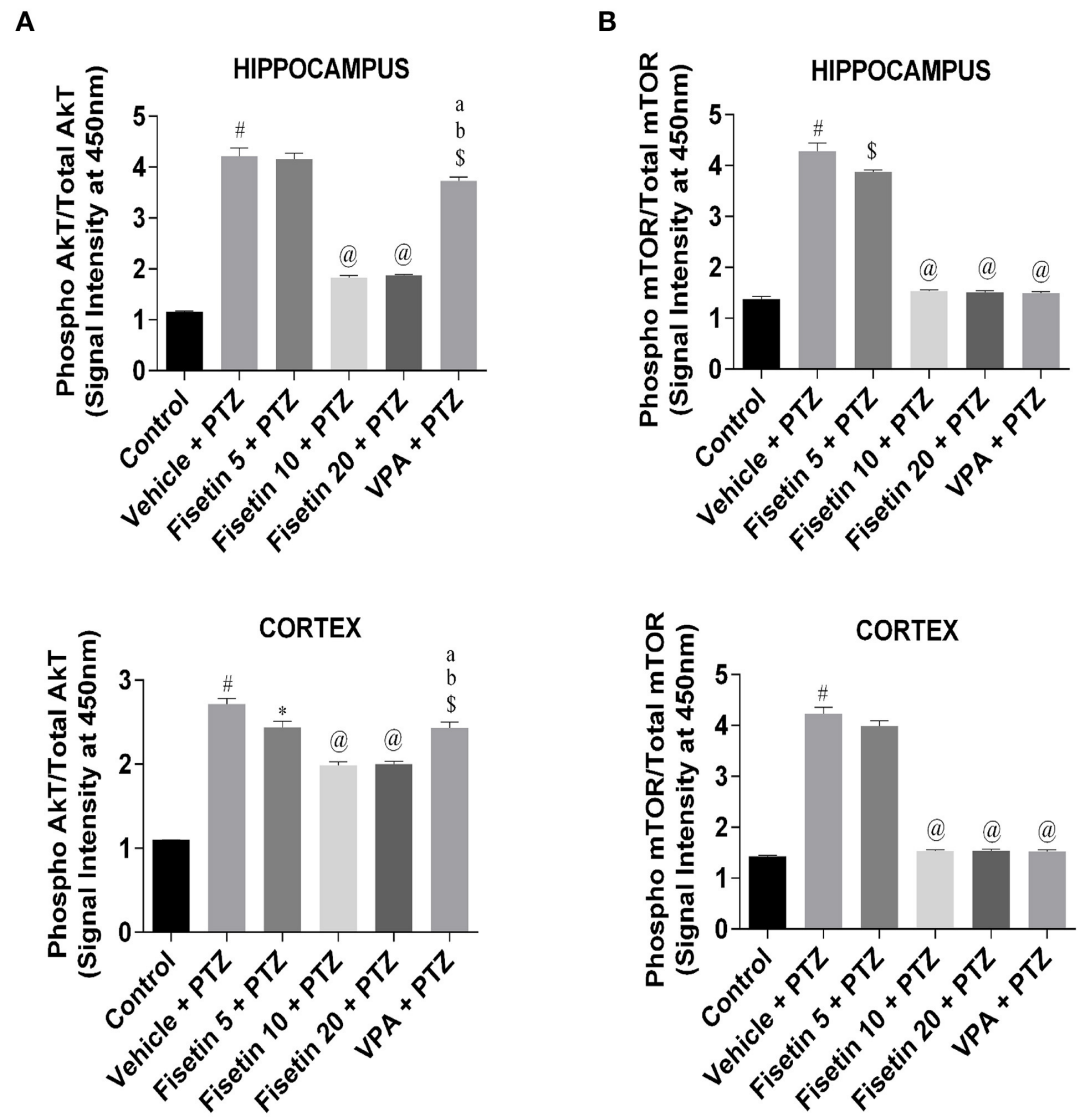
Effect of fisetin administration on AkT/mTOR phosphorylation in kindled mice. Phosphorylation of AkT and mTOR was measured by phosphorylation ELISA. **(A)** Phosphorylation of AkT in hippocampus and cortex. **(B)** Phosphorylation of mTOR in hippocampus and cortex. Compared with the control group, the phosphorylation of AkT and mTOR was increased in vehicle + PTZ group. PTZ-induced activation of AkT/mTOR was suppressed by fisetin administration in a dose-dependent manner. Data were analyzed by one-way ANOVA followed by Tukey's multiple comparison test (*n* = 6) and expressed as mean ± SEM. ^#^*p* < 0.001 as compared with control; ^*^p < 0.05 as compared with vehicle + PTZ group; ^$^*p* < 0.01 as compared with vehicle + PTZ group; ^@^*p* < 0.001 as compared with vehicle + PTZ group ^a^*p* < 0.001 as compared with fisetin 10 + PTZ group, and ^b^*p* < 0.001 as compared with fisetin 20 + PTZ group.

Phosphorylation ELISA kits were used to estimate the activation of AkT/mTOR cascade (Figures 7A,B; Supplementary Tables 10, 11) in the hippocampus and cortex tissues of the experimental mice. The ratio of phospho AkT/total AkT and phospho mTOR/total mTOR in both hippocampus and cortex was significantly upregulated (*p* < 0.001) in the vehicle + PTZ group as compared with the control group, showing the activation of AkT/mTOR cascade as a consequence of PTZ insult. However, fisetin administration in the dose of 5 mg/kg in the kindled mice failed to show any effect in the hippocampus, but it significantly lowered (*p* < 0.05) the phospho AkT/total AkT ratio in the cortex as compared with the vehicle + PTZ group. The fisetin 5 mg/kg, p.o. + PTZ group was able to suppress phospho mTOR/total mTOR in the hippocampus in a significant manner (*p* < 0.01) as compared with the vehicle + PTZ group but failed to show any effect in the cortex. Treatment with fisetin (10 and 20 mg/kg, p.o.) in the kindled mice significantly lowered (*p* < 0.001) the ratio of phospho AkT/total AkT and phospho mTOR/total mTOR in both hippocampus and cortex of the kindled mice in comparison with the vehicle + PTZ group. Further, in the VPA + PTZ group, the phosphorylation of AkT and mTOR was significantly decreased in both hippocampus and cortex (*p* < 0.01 and *p* < 0.001, respectively) as compared with the vehicle + PTZ group. Also, the phosphorylation of AkT in the VPA + PTZ group was significantly increased (*p* < 0.001) as compared with the fisetin 10 mg/kg, p.o.+ PTZ and fisetin 20 mg/kg, p.o. + PTZ groups. The results suggest that fisetin inhibits AkT/mTOR phosphorylation, which may be responsible for its anti-inflammatory mechanism as this pathway is involved in cytokine production in neuronal cells.

### Effect of Fisetin Administration on Cytochrome C and Caspase-3 in the Hippocampus and Cortex of Kindled Mice

**Figure 8:**
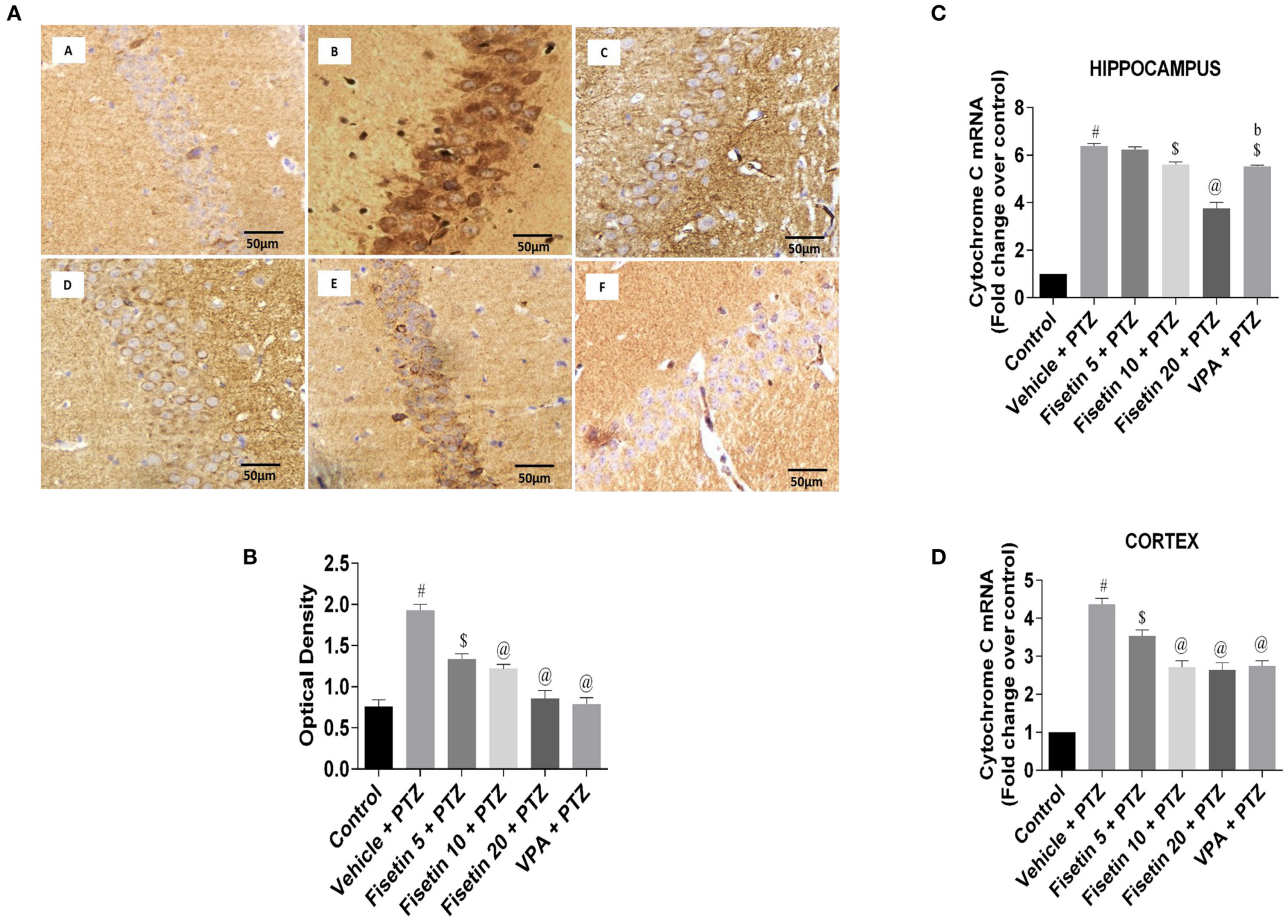
Effect of fisetin administration on apoptosis marker, cytochrome C in kindled mice. **(A)** Photomicrographs of the hippocampus in different experimental groups are shown (40X). (A) Control, (B) vehicle + PTZ, (C) fisetin 5 + PTZ, (D) fisetin 10 + PTZ, (E) fisetin 20 + PTZ, and (F) VPA + PTZ. Fisetin administration resulted in reduced immunoreactivity of cytochrome C protein in pyramidal cells of the hippocampus compared with the vehicle + PTZ group. **(B)** Quantitative estimation of cytochrome C immunostaining (*n* = 3). **(C)** mRNA expression of cytochrome C in the hippocampus (*n* = 5). **(D)** mRNA expression of cytochrome C in the cortex (*n* = 5). Data were analyzed by one-way ANOVA followed by Tukey's multiple comparison test and expressed as mean ± SEM. ^#^*p* < 0.001 as compared with control group; ^$^*p* < 0.01 as compared with vehicle + PTZ group; ^@^*p* < 0.001 as compared with vehicle + PTZ group, and ^b^*p* < 0.001 as compared with fisetin 20 + PTZ group.

Figures 8A,B (Supplementary Figure 4; Supplementary Table 12) represent the IHC staining of cytochrome C in the hippocampus of the experimental mice. The vehicle + PTZ group showed increased optical density indicative of significantly increased (*p* < 001) cytochrome C immunoreactivity in the hippocampus as compared with the control group. The fisetin 5 mg/kg, p.o. +PTZ group showed significantly reduced (*p* < 0.01) expression of cytochrome C, depicted by lesser immunoreactivity relative to the vehicle + PTZ group. The fisetin 10 mg/kg, p.o. + PTZ, fisetin 20 + PTZ, and VPA + PTZ groups showed significantly decreased (*p* < 0.001) immunoreactivity in the CA1 region of the hippocampus as compared with the vehicle + PTZ group. Furthermore, fisetin administration resulted in lowering of mRNA expression of cytochrome C in the hippocampus and cortex of the kindled mice in a dose-dependent manner (Figures 8C,D; Supplementary Table 12).

**Figure 9:**
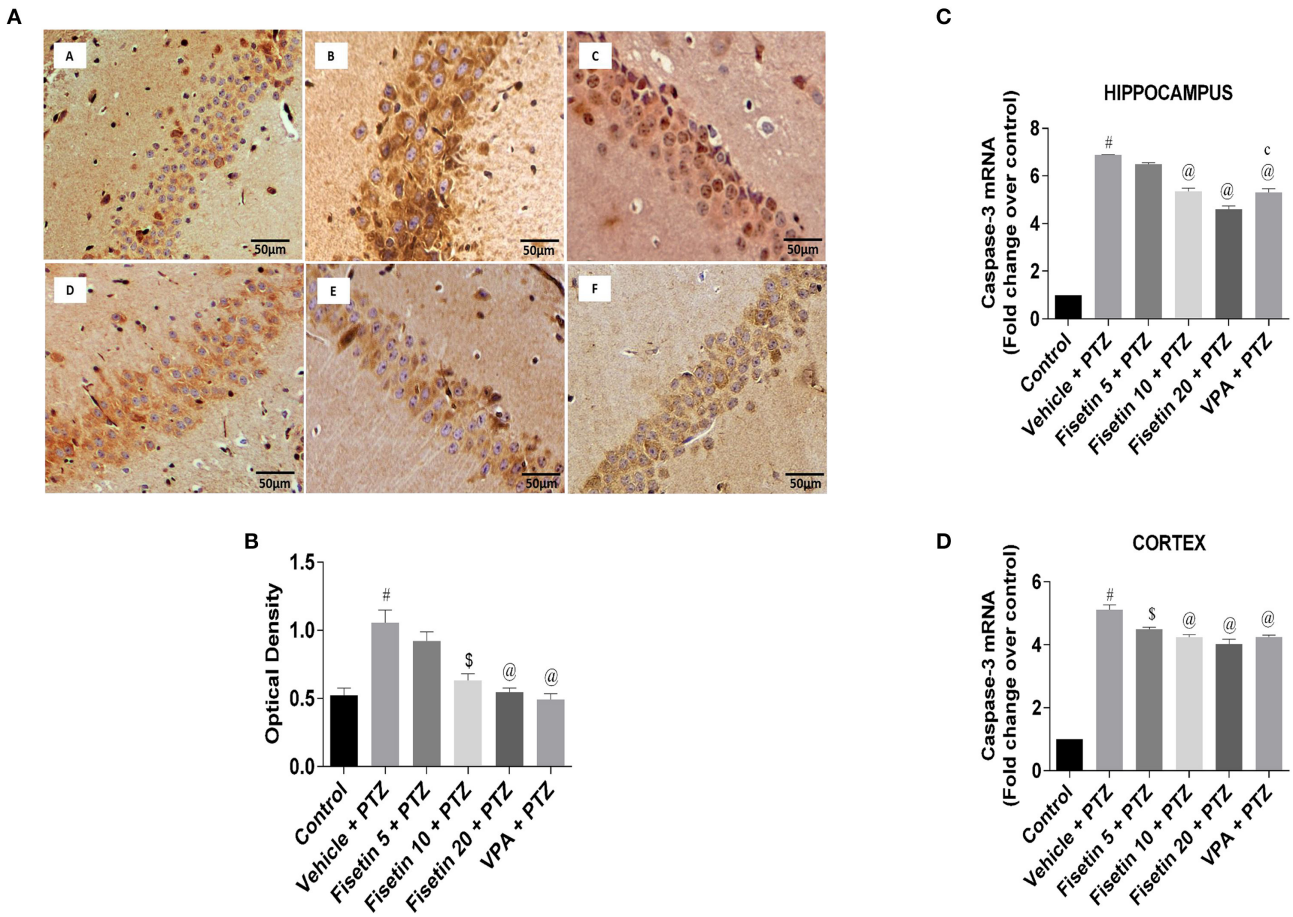
Effect of fisetin administration on apoptosis marker, caspase-3 in kindled mice. **(A)** Photomicrographs of the hippocampus in different experimental groups are shown (40X). (A) Control, (B) vehicle + PTZ, (C) fisetin 5 + PTZ, (D) fisetin 10 + PTZ, (E) fisetin 20 + PTZ, and (F) VPA + PTZ. Fisetin administration resulted in reduced immunoreactivity of caspase-3 protein in pyramidal cells of the hippocampus compared with the vehicle + PTZ group. **(B)** Quantitative estimation of caspase-3 immunostaining (*n* = 3). **(C)** mRNA expression of caspase-3 in the hippocampus (*n* = 3). **(D)** mRNA expression of caspase-3 in the cortex (*n* = 3). Data were analyzed by one-way ANOVA followed by Tukey's multiple comparison test and expressed as mean ± SEM. ^#^*p* < 0.001 as compared with control group; ^$^*p* < 0.01 as compared with vehicle + PTZ group; ^@^*p* < 0.001 as compared with vehicle + PTZ group, and ^c^*p* < 0.01 as compared with fisetin 20 + PTZ group.

Figures 9A,B (Supplementary Figure 5; Table 13) represent the IHC staining of caspase-3 in the hippocampus of the experimental groups. The optical density in the vehicle + PTZ group was significantly increased (*p* < 0.001) as compared with the control group, indicative of intense caspase-3 immunostaining. The fisetin 10 mg/kg, p.o. + PTZ, fisetin 20 mg/kg, p.o. + PTZ, and VPA + PTZ groups showed significantly lower immunoreactivity of caspase-3 (*p* < 0.01, *p* < 0.001, and *p* < 0.001, respectively) as compared with the vehicle + PTZ group. Figures 9C,D (Supplementary Table 13) represent the mRNA expression of caspase-3 in the hippocampus and cortex of the experimental mice. Caspase-3 mRNA expression in the hippocampus and cortex of the vehicle + PTZ group upregulated significantly (*p* < 0.001) as compared with control. The fisetin 5 mg/kg, p.o. + PTZ group did not show any significant decrease in the caspase-3 mRNA level in the hippocampus but was significantly lower in the cortex (*p* < 0.01) as compared with the vehicle + PTZ group. However, the fisetin 10 mg/kg, p.o.+ PTZ, fisetin 20 mg/kg, p.o. + PTZ, and VPA + PTZ groups reported significant downregulation (*p* < 0.001) of the caspase-3 mRNA expression in the hippocampus and cortex as compared with the vehicle + PTZ group. These findings support the apoptosis-suppressing mechanism of fisetin in a dose-dependent manner contributing to its neuroprotective efficacy.

## Discussion

Fisetin has been reported to possess anti-inflammatory and disease-modifying effects on several brain conditions, such as epilepsy, synaptic dysfunction, intracerebral hemorrhage, and diabetic neuropathy (12, 40, 41, 52). Therefore, this study was designed to explore the potential effect of fisetin during epileptogenesis.

In this study, fisetin administration increased the STC in the ICES test when compared with the control group in a dose-dependent manner. Similarly, a previous study has reported the neuroprotective effect of fisetin in maximal electroshock seizures and electrical kindling in rats (41), which supports that fisetin has the potential to prevent electrically-induced seizures. This study showed that fisetin administration in doses 10 and 20 mg/kg, p.o. has increased the latency to MJ and GS in PTZ-induced seizures. A previous study had reported that fisetin administration resulted in the reduction of seizure severity and mortality in PTZ-induced seizures in experimental mice (41). Hence, the findings of this study are in line with previous studies that have highlighted the anti-seizure effect of fisetin. The treatments that are effective against tonic hind limb extension induced by electroshock are effective against partial and generalized tonic–clonic seizures (53), and those showing efficacy in the PTZ model are effective against absence seizures (54). The observed effects in these models indicate that fisetin has the potential anti-seizure effect against both generalized and absence seizures.

Chemical kindling induced by repetitive injections of PTZ at a sub-convulsive dose (25 mg/kg, i.p.) leads to the generation of seizures that simulate clinical absence epilepsy and myoclonic, generalized tonic–clonic (primary generalized) seizures (55). PTZ-induced kindling model is the preferred approach for studying neurochemical and long-term structural alterations in the brain accompanying epileptogenesis, and is widely accepted for the development of potential ASDs (55). In this study, fisetin administration resulted in (i) delayed onset of seizures; (ii) decreased the percentage of fully kindled mice; (iii) attenuated seizure severity score; and (iv) decreased mortality in kindled mice. The findings of this study are in concordance with an earlier study in which treatment with flavonoid molecule from *Morus alba* led to suppression of seizure severity in rats (56). Similarly, naringin, a flavonoid, was reported to have an anti-seizure effect against PTZ-induced kindling in Wistar rats (57).

The pyramidal cells of the CA1 hippocampal region are particularly sensitive to various cytotoxic insults, which are reflected in neuronal necrosis of the area (58). Moreover, neuronal damage in the hippocampal CA1 region is a common pathological finding in patients with epilepsy (59). Neuronal damage associated with PTZ-induced kindling may be a possible consequence of recurrent seizure activity (60). In this study, histopathological results from Nissl staining showed significant neuronal damage in the CA1 area in the PTZ group, which is in agreement with previous studies (61, 62). However, fisetin administration provided significant protection against hippocampal neuronal damage in a dose-dependent manner. The protective effect of fisetin against hippocampal neuronal loss has been previously reported in lipopolysaccharide-induced neurodegeneration in adult mice (63). Another study revealed that hippocampal neurodegeneration was ameliorated by fisetin administration in Aβ_1−42_-induced neurotoxicity (40). Furthermore, a study documented that fisetin administration was able to restore morphological dysfunction in the pyramidal cells of the hippocampus in aluminum chloride-induced neurotoxicity in mice (35).

Dysregulated neuroinflammatory response has a pivotal role in several CNS conditions, such as acute and chronic neurodegenerative disorders, TBI, and epilepsy. Ineffective anti-inflammatory control by endogenous mechanisms may play a key role in igniting persisting neuroinflammation. Several pre-clinical and clinical evidence points toward the involvement of inflammatory mediators in the pathogenesis of seizures and associated comorbidities in epilepsy. Moreover, inflammatory mediators are endowed with CNS-specific neuromodulatory roles that may contribute to hyperexcitability and excitotoxicity (64).

NF-κB, a transcription factor, initiates and regulates the expression of several inflammatory mediators such as COX-2, which has a crucial role in upregulating cytokine expression (65). Also, COX-2 is a central link between various inflammatory cascades and contributes to seizure generation and development of epilepsy (66). In this study, the vehicle + PTZ group exhibited significant induction of NF-κB and COX-2 in the hippocampus and cortex of the mice. This is in line with the previously reported studies where PTZ kindling resulted in high expression of NF-κB and COX-2 in rodents (67, 68). Fisetin administration was able to impede the expression of NF-κB and COX-2 in the hippocampus and cortex of kindled mouse brain, suggesting the probable anti-inflammatory effect of fisetin that may contribute to its neuroprotective effect against seizure severity. Previous investigations using a diabetic neuropathy model showed that fisetin markedly inhibited NF-κB signaling with simultaneous influence on Nrf2 (52). Similarly, the effect of fisetin against intracerebral hemorrhage-induced neuroinflammation was also reported to be modulated *via* mediators of the NF-κB pathway in aged mice (12). Moreover, fisetin had also shown anti-inflammatory activity *via* the inactivation of NF-κB in macrophage cells (11).

IL-1R1/TLR-4 signaling is a key upstream generator of the neuroinflammatory response. Its activation by endogenous ligands leads to the transcriptional induction of NF-κB–regulated inflammatory genes, and, as a consequence, to the generation and rapid amplification of the inflammatory cascade (69). The IL-1R1/TLR-4 axis is rapidly and persistently activated in brain areas involved in seizure generation and propagation (70). Such changes do not necessarily reflect ongoing seizure activity, since they also occur before the beginning of spontaneous seizures, implying their potential involvement in ictogenesis. Interestingly, a study reported that pharmacological targeting of IL-1β by administering IL-1R1 antagonist reduced seizure susceptibility in an experimental rodent model of TBI (71). In addition, increased levels of proinflammatory cytokines IL-1β, TNF-α, HMGB1, IL-6, and downstream inflammatory cascade have also been reported in surgically resected brain tissue samples of patients (72). A study demonstrated that both IL-1β and HMGB1 may play a role in lowering the ictal event threshold (70). Moreover, anti-HMGB1 monoclonal antibody exhibited decreased seizure severity in an experimental seizure model by downregulating the levels of neuroinflammatory molecules, such as TLR-4 and IL-6, in the hippocampus and cortex (73). Furthermore, TLR-4 being the major receptor for HMGB1 is a molecule of interest for assessing the pharmacological activity of potential ASDs (74). In this study, we observed increased levels of HMGB1 and TLR-4 in the vehicle + PTZ group, suggesting its role in epileptogenesis, which is in line with the previous studies where PTZ kindling resulted in increased mRNA expression of HMGB1 and TLR-4 in brain tissues of mice and zebrafish (75, 76). Furthermore, the administration of fisetin suppressed the levels of HMGB1 and TLR-4 in the hippocampus and cortex of the kindled mice in a dose-dependent manner. This investigation supports that the HMGB1/TLR-4 axis is contributing toward seizure mechanisms during epilepsy (13). In a study, it was reported that the IL-1R1 expression and IL-1β levels were elevated in a rodent model of PTZ-induced kindling (77). In line with this study, our study showed increased levels of IL-1β and IL-1R1 in the hippocampus and cortex regions of the vehicle + PTZ group. Interestingly, fisetin administration decreased the levels of IL-1β and IL-1R1 in the hippocampus and cortex of the kindled mice in a dose-dependent manner, proposing its potential anti-inflammatory mechanisms in the brain.

TNF-α is another endogenous cytokine generated during the process of epileptogenesis. Studies reported that levels of TNF-α are found to be raised after seizure activity in rodents and clinical subjects (78). Paradoxically, another study reported that TNF-α injection in the hippocampus reduces seizures mediated by neuronal p75 receptors in mice (79). However, in this study, we observed a significant increase in the levels of TNF-α in the vehicle + PTZ group. Fisetin administration significantly decreased the levels of TNF-α in the hippocampus and cortex of the kindled mice. The observed anti-inflammatory effect of fisetin is in accordance with the previous report, which suggests that fisetin protects against lipopolysaccharide-induced activation of inflammatory mediators, such as TNF-α and IL-1β (63).

IL-6, a key inflammatory molecule, plays a pivotal role in the progress of the excitatory mechanism of the brain and is well-connected with the development of generalized seizures (80). In this study, an increased level of IL-6 was observed in the vehicle + PTZ group, which is in consensus with an earlier study conducted in our laboratory and by other researchers (20, 81). The findings of this study showed that fisetin administration in the kindled mice decreased the level of IL-6, indicating its neuroinflammation-controlling mechanism, which might play a role in its anti-seizure efficacy. This is in agreement with a study where fisetin administration has decreased IL-6 levels in the brain tissue of an animal model of Parkinson's disease (82).

Besides, several other signaling cascades are interrupted after neuronal excitation during seizures and have been a target of interest for treatment against epilepsy (83). Specifically, the mTOR signaling cascade is upregulated in patients with epilepsy, as well as in several *in vivo* genetic and acquired experimental models of epilepsy (84). Immune signaling and the mTOR pathway are reported to interact both under normal physiological conditions and in the diseased state. Activation of mTOR signaling is critical for the cellular processes of immune cells in the CNS. Also, the inhibition of mTOR activity resulted in impaired maturation and function of dendritic cells (DCs) and inhibited T-cell proliferation (85), suggesting its importance in controlling inflammation. Upregulation of mTOR signaling also influences cytokine production and release, especially in DCs. We observed a significant increase in brain AkT/mTOR phosphorylation that was allied with increased levels of inflammatory cytokines in the vehicle + PTZ group. However, treatment with fisetin suppressed the phosphorylation of AkT/mTOR in a dose-dependent manner. Similarly, resveratrol, a natural polyphenolic compound, has been shown to have antiseizure effects on epilepsy models, such as status epilepticus, when it was administered before the induction of seizures (86) attributed to its ability to suppress proinflammatory cytokines TNF-α, which was reported to be regulated by mTOR cascade (87). In another study, resveratrol pre-administration decreased the expression of phospho-mTOR and inflammatory mediators, such as NF-κB, IL-1β, and COX-2, following seizures, suggesting that anti-inflammatory property is mediated by mTOR signaling (88). Thus, these polyphenolic compounds have the ability to inhibit mTOR signaling, which may have a possible role in their anti-inflammatory mechanism.

Mitochondrial dysfunction during seizures allows translocation of proapoptotic molecules, which permits the diffusion of cytochrome C release from mitochondria to the cytoplasm where it activates caspase-9, which further leads to activation of caspase-3 (89). Apoptotic proteins like BAD-BAX, caspase-3 can regulate neuroplastic changes independent of cell death in the CNS and contribute to epileptogenic processes (90, 91). This study showed a significant increase in the expression of cytochrome C and caspase-3 in the brain regions, which is in concordance with reported studies (92, 93). In addition, neuronal damage associated with PTZ kindling is partly due to the upregulation of apoptotic mediators (93). However, in this study, fisetin administration was able to suppress apoptotic molecules in the hippocampus and cortex of the PTZ-kindled mice. The anti-apoptotic property of fisetin was earlier demonstrated by increased expression of B-cell lymphoma 2 (Bcl-2), while decreased expression of Bcl-2-associated X protein (Bax) and caspase-3 in the experimental TBI model (36). In addition, fisetin ameliorated apoptotic signals by preventing the release of cytochrome C and downregulating the expression of caspase-3 and MAPK-1 in developmental methylmercury neurotoxicity (34). In a recent study, fisetin showed neuroprotection in rotenone-induced toxicity in the cellular model of Parkinson's disease by inhibiting the expression of Bax and caspase-3, and upregulating the expression of Bcl-2 (94). In accordance, the present study showed that fisetin administration in kindled mice was able to downregulate cytochrome C and caspase-3 expression, which might be a possible mechanism for its anti-seizure activity.

## Conclusion

The findings from this study have shown that the already proven neuroprotective effect of fisetin due to its anti-inflammatory and antiapoptotic properties can be used to inhibit the underlying pathology of epileptogenesis. Fisetin can be further studied for its anti-inflammatory mechanism, which can pave the way for newer research areas in treating various other neurological diseases. In this study, the administration of fisetin by oral route showed anti-seizure activity in various models of epilepsy by modifying the underlying mechanisms. However, further research is required to confirm the disease-modifying effect of fisetin by targeting inflammatory and apoptotic cascades.

## Data Availability Statement

The original contributions presented in the study are included in the article/Supplementary Material, further inquiries can be directed to the corresponding author.

## Ethics Statement

The animal study was reviewed and approved by Institutional Animal Ethics Committee—(Reg. No. and Date of Reg: 173/GO/Re/S/2000/CPCSEA, 28th January 2000), Jamia Hamdard, New Delhi, India.

## Author Contributions

SK: investigation, resources, formal analysis, methodology, writing original draft, and data curation. NA: conceptualization, methodology, resources, validation, formal analysis, writing—review editing, visualization, and supervision. MS: visualization and supervision. OA: methodology and visualization. All authors contributed to the article and approved the submitted version.

## Conflict of Interest

The authors declare that the research was conducted in the absence of any commercial or financial relationships that could be construed as a potential conflict of interest.
