# Supplementary material for: Neuroprotective Effect of Fisetin Through Suppression of IL-1R/TLR Axis and Apoptosis in Pentylenetetrazole-Induced Kindling in Mice
Source: Front Neurol. 2021 Jul 21;12:689069. doi: 10.3389/fneur.2021.689069 (PMC8333701; doi:10.3389/fneur.2021.689069)
Supplement: Supplementary file 14 [file Data_Sheet_1.PDF]

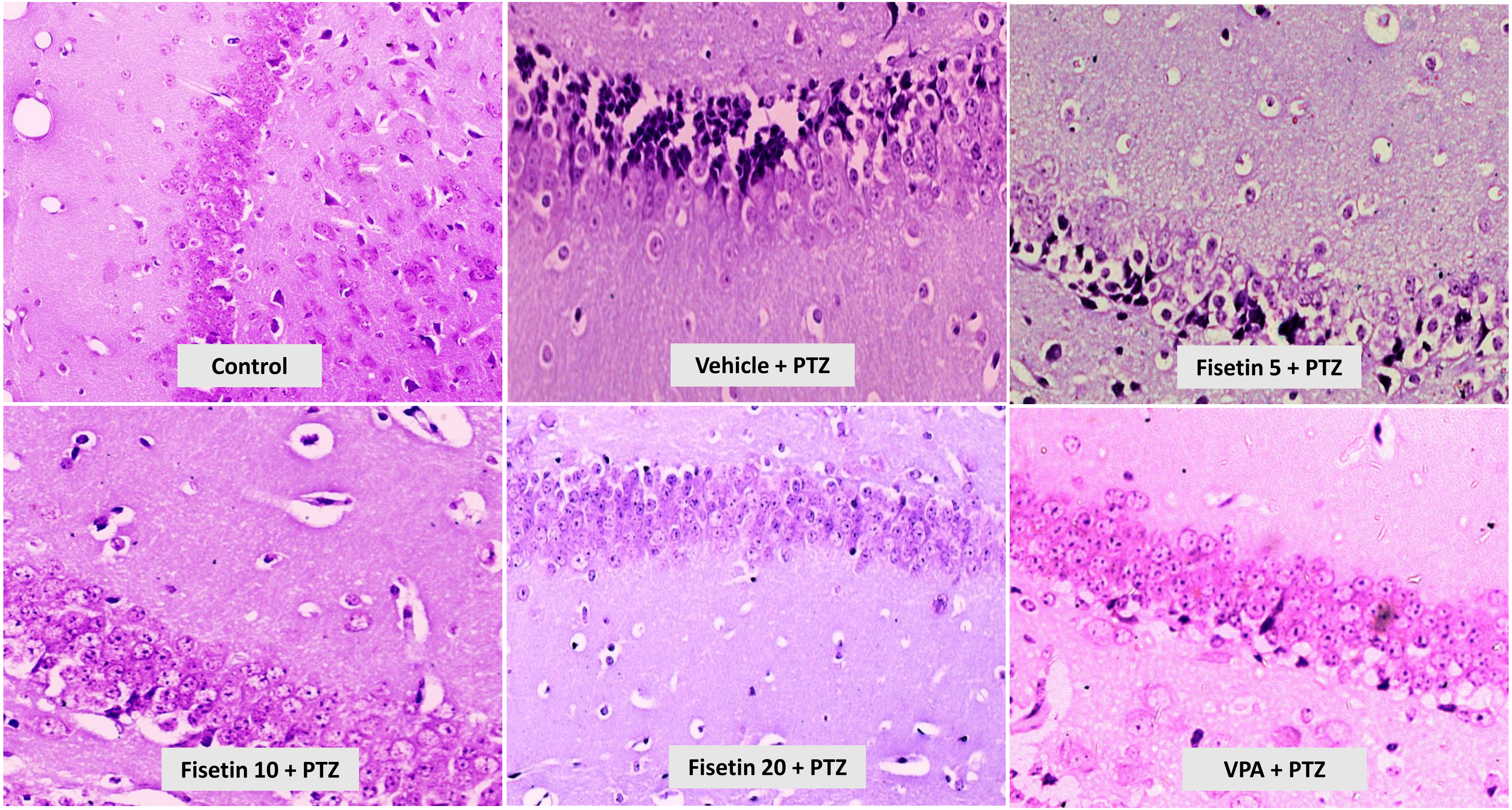

FIGURE (1): NISSL STAINING

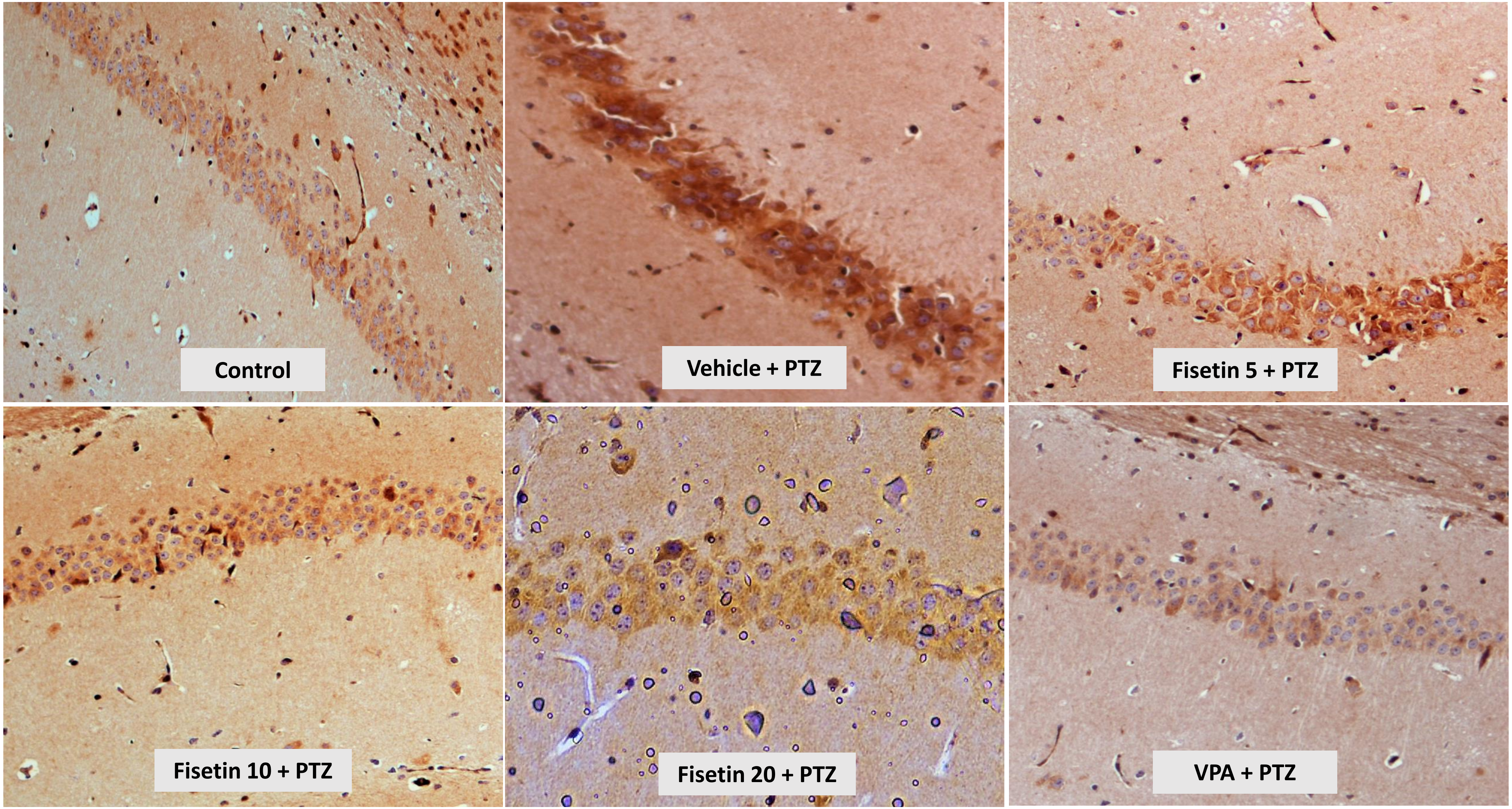

FIGURE (2): IMMUNOHISTOCHEMISTRY OF NF-κB

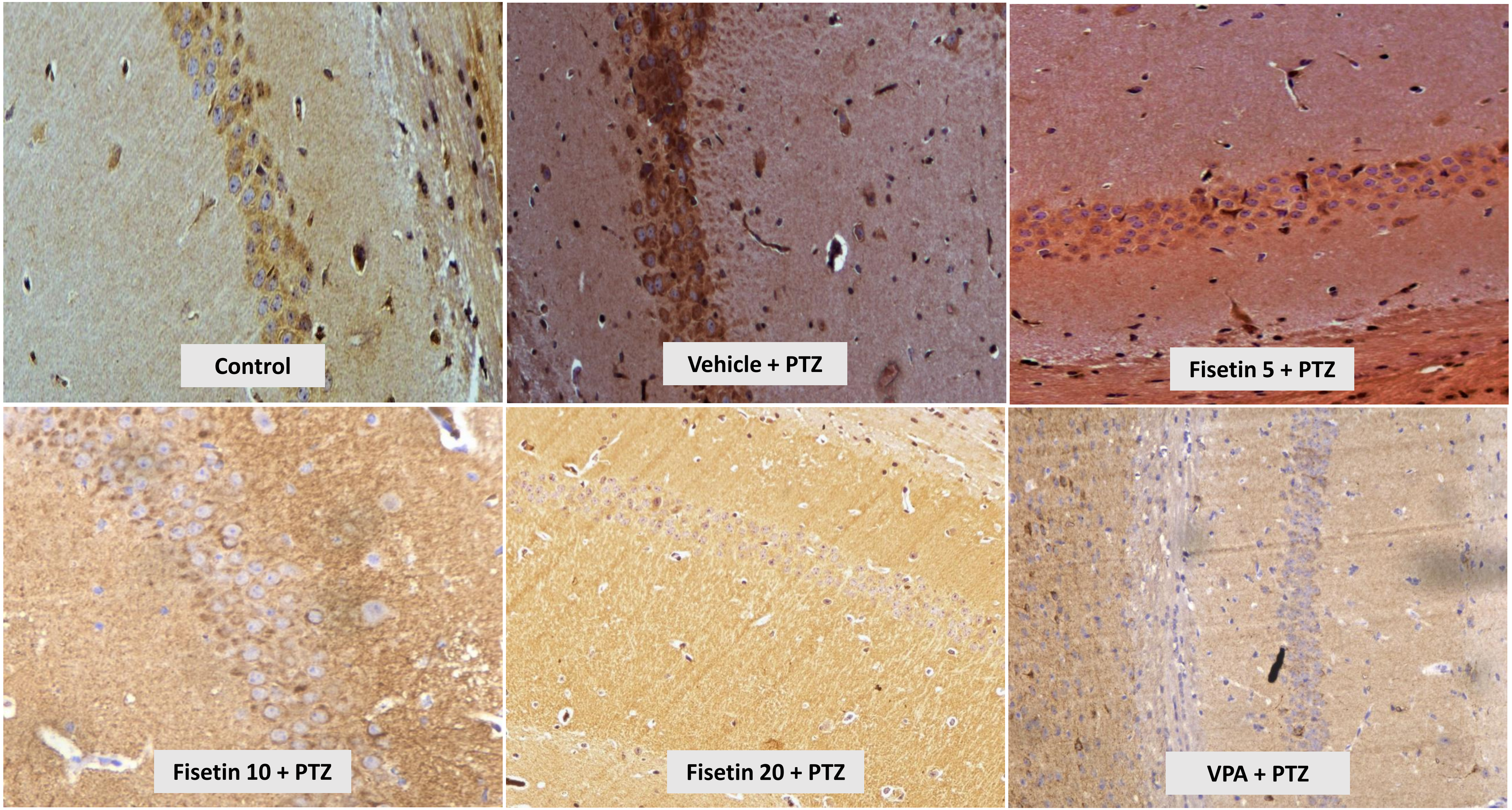

FIGURE (3): IMMUNOHISTOCHEMISTRY OF COX-2

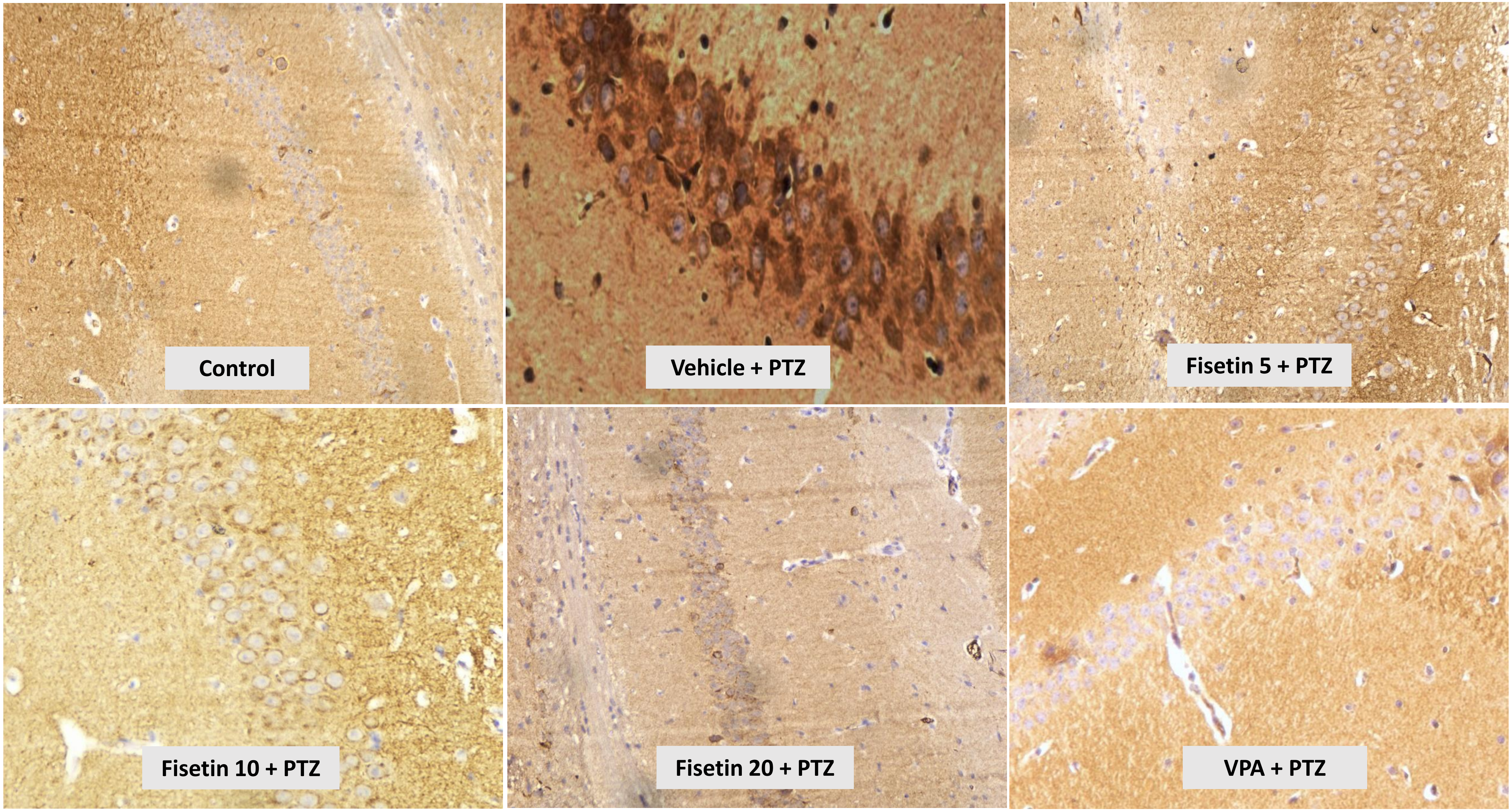

FIGURE (4): IMMUNOHISTOCHEMISTRY OF Cytochrome C

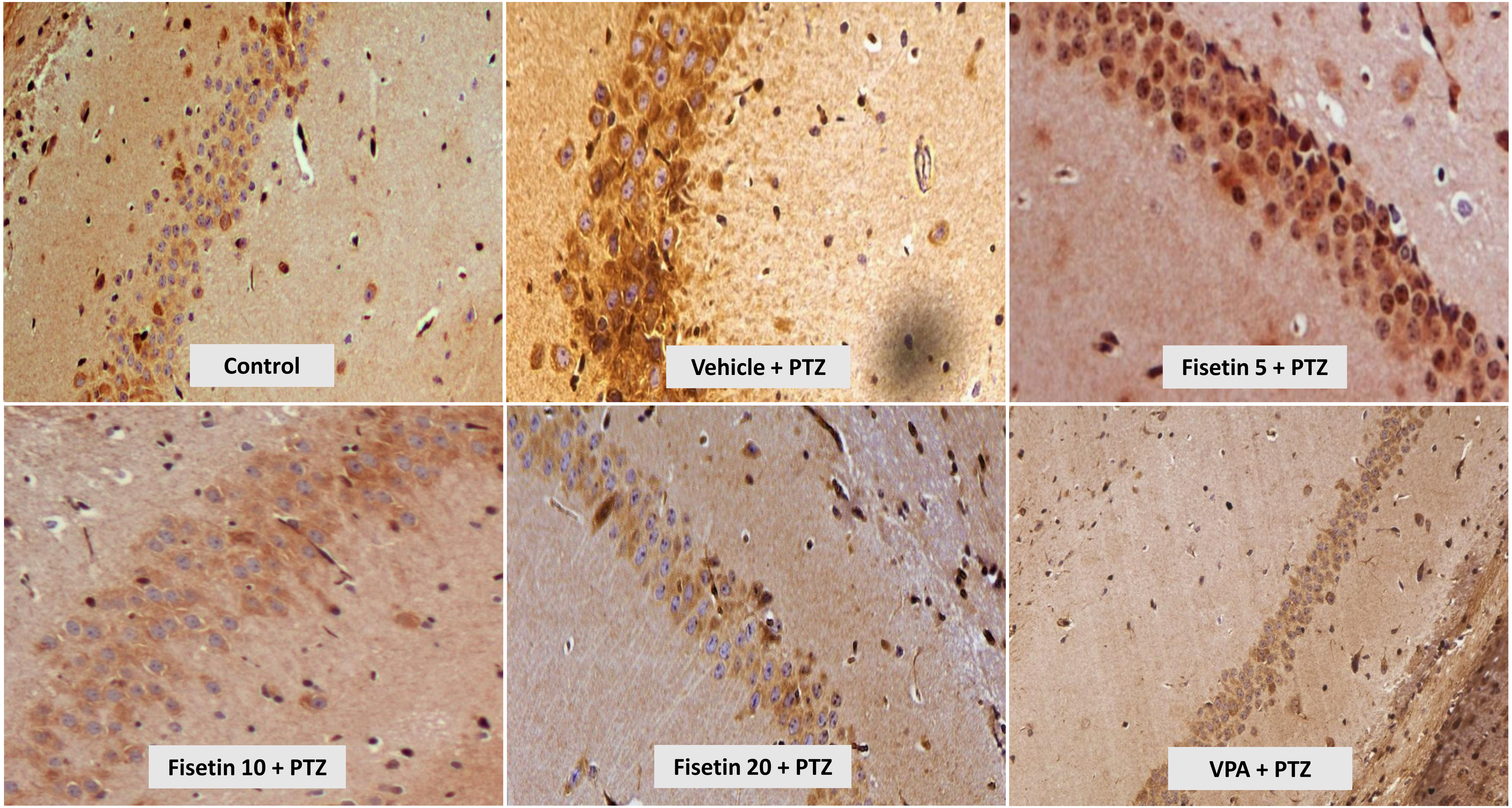

FIGURE (5): IMMUNOHISTOCHEMISTRY OF Caspase-3
